# Regeneration and Degradation in a Biomimetic Polyoxometalate Water Oxidation Catalyst

**DOI:** 10.1021/acscatal.2c06301

**Published:** 2023-02-14

**Authors:** Ludwig Schwiedrzik, Tina Rajkovic, Leticia González

**Affiliations:** † Institute of Theoretical Chemistry, Faculty of Chemistry, University of Vienna, Währinger Straße 17, 1090 Vienna, Austria; ‡ Vienna Doctoral School in Chemistry (DoSChem), University of Vienna, Währinger Str. 42, 1090 Vienna, Austria

**Keywords:** artificial photosynthesis, polyoxometalate, Jahn−Teller axis, electrocatalysis, regeneration, degradation, density functional theory

## Abstract

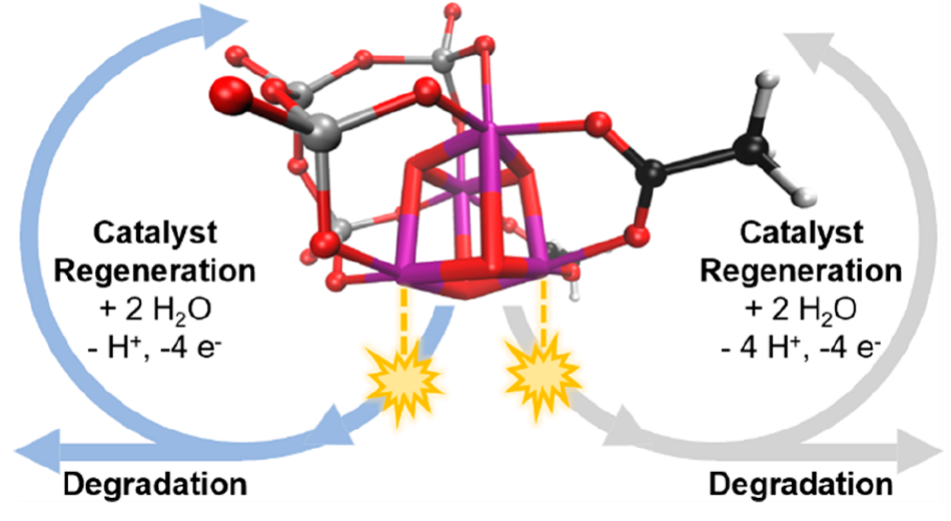

Complete understanding of catalytic cycles is required to advance the design of water oxidation catalysts, but it is difficult to attain, due to the complex factors governing their reactivity and stability. In this study, we investigate the regeneration and degradation pathways of the highly active biomimetic water oxidation catalyst [Mn^3+^_2_Mn^4+^_2_V_4_O_17_(OAc)_3_]^3–^, thereby completing its catalytic cycle. Beginning with the deactivated species [Mn^3+^_4_V_4_O_17_(OAc)_2_]^4–^ left over after O_2_ evolution, we scrutinize a network of reaction intermediates belonging to two alternative water oxidation cycles. We find that catalyst regeneration to the activated species [Mn^4+^_4_V_4_O_17_(OAc)_2_(OH)(H_2_O)]^−^ proceeds via oxidation of each Mn center, with one water ligand being bound during the first oxidation step and a second water ligand being bound and deprotonated during the final oxidation step. ΔΔ*G* values for this last oxidation are consistent with previous experimental results, while regeneration within an alternative catalytic cycle was found to be thermodynamically unfavorable. Extensive in silico sampling of catalyst structures also revealed two degradation processes: cubane opening and ligand dissociation, both of which have low barriers at highly reduced states of the catalyst due to the presence of Jahn–Teller effects. These mechanistic insights are expected to spur the development of more efficient and stable Mn cubane water oxidation catalysts.

## 1 Introduction

Humanity’s overreliance on fossil fuels is the main driver of the current climate and energy crises, the effects of which are increasingly being felt across the globe.^1−3^ Searching for alternatives, researchers have worked for decades to develop new technologies for converting solar to chemical energy.^4,5^ Among these, artificial water splitting plays a prominent role, promising to create renewable H_2_ from water and sunlight.^6,7^ Water splitting is a four-electron redox reaction consisting of two half-reactions: water oxidation and hydrogen evolution. Since water oxidation is considered the more challenging of the two, great efforts have been made to develop efficient water oxidation catalysts.^7−13^ Inspired by the oxygen-evolving complex (OEC), which contains the tetramanganese cubane active center responsible for water oxidation in natural photosynthesis, a large number of biomimetic catalysts featuring four metal centers in a cubane arrangement have been synthesized and investigated.^13−28^

Among such systems, those featuring Co_4_O_4_ and Mn_4_O_4_ cubane cores have been intensively studied, often as model systems for the natural OEC or for heterogeneous catalysts relevant for industrial-scale water splitting. In this context, the cubanes’ ability to flexibly redistribute electrons between metal centers has been leveraged to explain their high water oxidation activity.^18−20^ Jahn–Teller (JT) effects have been noted to significantly alter the structure^29−37^ and even the reactivity of Mn-containing catalysts featuring Mn^3+^ centers.^38−41^ Such distortions are present in d^4^ metal centers such as Mn^3+^ and lead to the elongation of one bond axis and concomitant shortening of the other two bond axes in an octahedral coordination environment. They represent a form of structural flexibility that, when taken together with the aforementioned facile electron redistribution, makes Mn-oxo cubane catalysts featuring Mn^3+^ centers particularly promising candidates for increasing the efficiency of the water oxidation reaction.^40−44^

**Figure 1:**
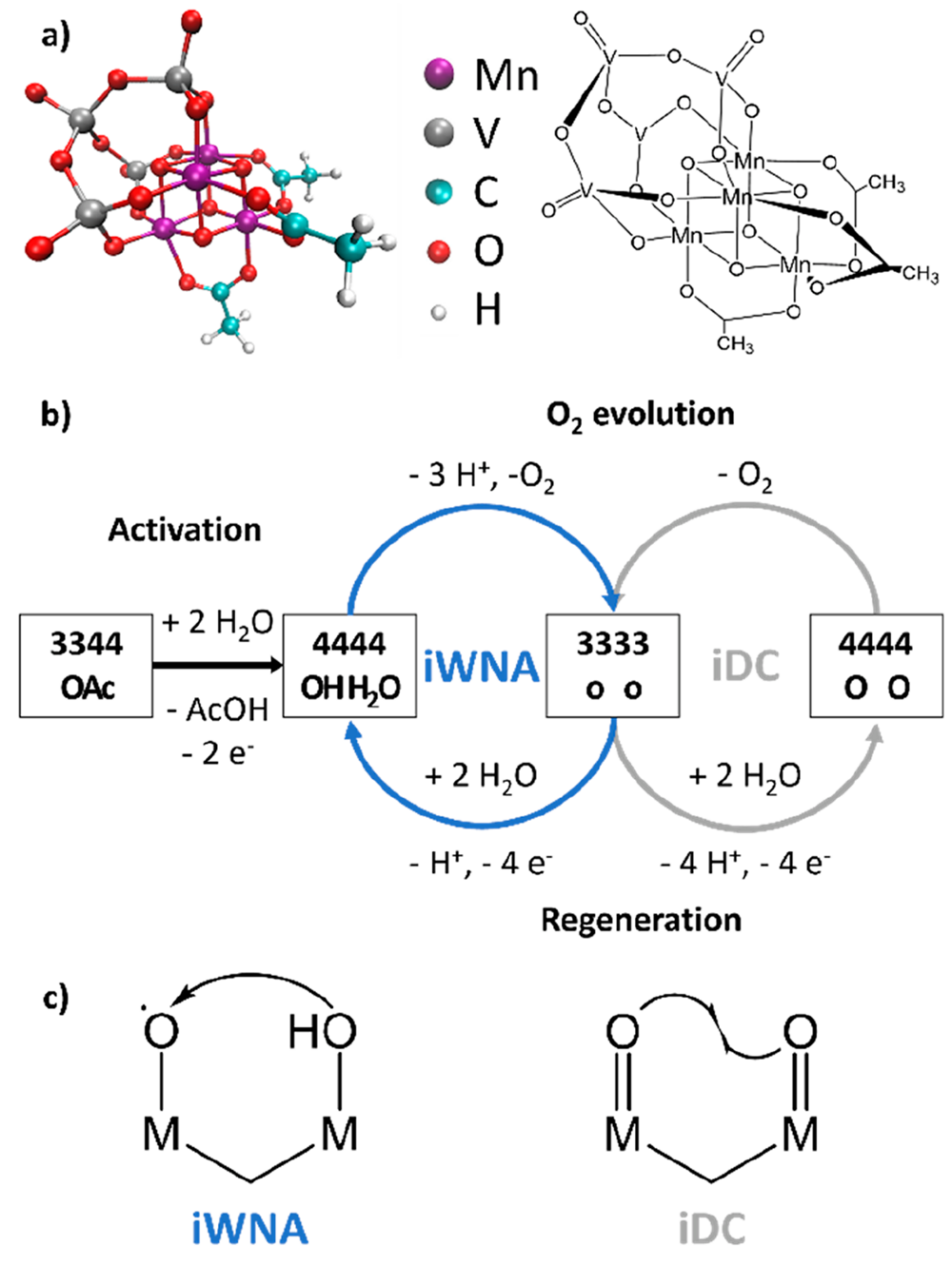
(a) Three-dimensional structure of [Mn^3+^_2_Mn^4+^_2_V_4_O_17_(OAc)_3_]^3–^ or **3344-OAc** with color legend (left) and its ChemDraw structure (right). (b) Reactivity scheme of **3344-OAc**, consisting of activation (black arrow), the proposed iWNA cycle between **4444-OH-H**_**2**_**O** and **3333-o-o** (blue), and the alternative proposed iDC cycle between **4444-O-O** and **3333-o-o** (gray). O_2_ evolution occurs in the upper half of each cycle, while the lower half corresponds to catalyst regeneration. (c) Different types of O–O bond formation mechanisms in multicenter water oxidation catalysts: intramolecular nucleophilic attack by an OH ligand on a neighboring terminal oxyl ligand (iWNA) and intramolecular direct coupling between two neighboring terminal oxo ligands (iDC).

In this work, we focus on the bioinspired water oxidation catalyst [Mn^3+^_2_Mn^4+^_2_V_4_O_17_(OAc)_3_]^3–^ (abbreviated as **3344-OAc**, where the numbers indicate the oxidation states of the Mn atoms; see Figure 1a).^45^ This highly active multicenter catalyst (turnover number (TON) > 12 000; turnover frequency (TOF) > 200 min^–1^)^46^ consists of an Mn_4_O_4_ cubane core, surrounded on three sides by a hexadentate V_4_O_13_ vanadate ligand and three bidentate acetate ligands on the remaining sides. A prior combined experimental and theoretical study showed that **3344-OAc** is actually a precatalyst that must first undergo activation by oxidation of two Mn centers to yield an Mn^4+^_4_ configuration of the cubane core as well as exchange of one acetate with an OH and an H_2_O ligand before the actual O_2_ evolution can be catalyzed (Figure 1b, black arrow). The activated species was determined to be [Mn^4+^_4_V_4_O_17_(OAc)_2_(OH)(H_2_O)]^−^ (**4444-OH-H**_**2**_**O**).^40^ Further theoretical work led to the proposal of a feasible mechanism for O_2_ evolution, consisting of three proton-coupled electron transfers (PCET) and one electron transfer (ET) step (Figure 1b, upper half of blue cycle).^41^ After O_2_ evolution, the catalyst was found to remain as [Mn^3+^_4_V_4_O_17_(OAc)_2_]^4–^ (**3333-o-o**), a deactivated species with two cofacial open coordination sites (**o**) that must undergo regeneration before being able to catalyze another turnover. The question of how **4444-OH-H**_**2**_**O** is regenerated from **3333-o-o** has thus far remained unanswered, leaving the understanding of the catalytic cycle incomplete.

In our proposed water oxidation cycle, O–O bond formation is achieved by the intramolecular attack of an OH ligand bound to one Mn center on a cofacial metal-oxyl group, as in the iWNA mechanism shown in Figure 1c.^41^ This is one variant of the so-called “water nucleophilic attack” (WNA) type of mechanism, which has been often invoked to explain the reactivity of the OEC, as well as that of a large number of synthetic water oxidation catalysts.^8,12,13,47,48^ In catalysts where multiple terminal metal-oxo groups are present in close proximity (including the OEC), an alternative form of O–O bonding has been proposed, coined a direct coupling (DC) type of mechanism—an intramolecular variant of which (iDC) is also shown in Figure 1c.^12,13,16,47,49,50^ While no species featuring two cofacial oxo or oxyl ligands have been invoked so far for our tetramanganese catalyst, this does not rule out that an iDC-type water oxidation cycle could exist alongside the previously proposed iWNA-type. We hypothesize that O–O bond formation according to the iDC mechanism could best be achieved by first binding water ligands at a low redox state, followed by combined deprotonation and metal-centered oxidation through a series of PCET steps (Figure 1b, lower half of gray cycle), leading to a highly oxidized catalyst with terminal oxo ligands, [Mn^4+^_4_V_4_O_17_(OAc)_2_(O)_2_]^4–^ (termed **4444-O-O**).

Previous work on **3344-OAc** paid particular attention to the role of JT effects in the reactivity of the catalyst. These effects have been noted to affect the structure and reactivity of the OEC as well as other synthetic catalysts containing d^4^ metal centers;^29−39^ JT effects have even been linked to increased water oxidation activity in heterogeneous catalysts.^42−44,51^ In **3344-OAc**, we found that JT effects play a pivotal role in multiple reaction steps. During activation, JT-elongated bonds provided key weak points for water attack and subsequent ligand exchange.^40^ We observed similar behavior for the O_2_ dissociation step of the water oxidation mechanism.^41^ Finally, we showed that during O_2_ evolution, a reorientation of JT-distorted bonds precedes the formation of the O–O bond, which is itself concerted with an ET from the reactive ligands to a Mn center and the emergence of JT distortions at that same metal center.^41^

The obvious importance of JT effects for the reactivity of **3344-OAc** motivated some of us to study in depth the relative stability of structures featuring different orientations of JT-distorted bonds across all relevant redox states of the catalyst, resulting in a set of heuristic rules for the comparison among such structures.^52^ In this context, we found that JT axes were energetically favorable when oriented toward the acetate or water ligands, but not toward the vanadate ligand, so that the vanadate appeared to stabilize the catalyst, which itself is overall quite flexible. The presence of a number of structures featuring open coordination sites as more stable alternatives to fully coordinated complexes at the lowest oxidation state of the catalyst (**3333**) was also noted and rationalized as an especially strong JT effect.^53^

In this work, we set out to investigate the regeneration mechanism of the **3344-OAc** catalyst from the deactivated **3333-o-o** form to the **4444-OH-H**_**2**_**O** one (lower half of the blue cycle in Figure 1b), formally
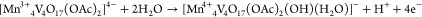
1by sampling the large number of structures that could be involved in that process. To this end, we propose a network of intermediates that connect the two species, featuring a variety of ligand configurations from the two open coordination sites of the deactivated species to the H_2_O and OH ligands of the activated catalyst. By comparing the stabilities of these various intermediates, we aim at uncovering a feasible regeneration mechanism for **3344-OAc**, thereby closing the proposed iWNA cyle. At the same time, we investigate the possibility of an iDC-type water oxidation cycle (shown in gray in Figure 1b). We focus on regeneration via binding of water ligands at a low oxidation state and a series of PCET steps, leading to a highly oxidized catalyst with terminal oxo ligands, **4444-O-O**, that is able to carry out O_2_ evolution according to the iDC mechanism. Finally, we expect that the exhaustive sampling of reaction intermediate structures here undertaken can shed further light on the role of JT effects in the reactivity and stability of **3344-OAc** and its derivatives.

## 2 Methods

### 2.1 Nomenclature

**Figure 2:**
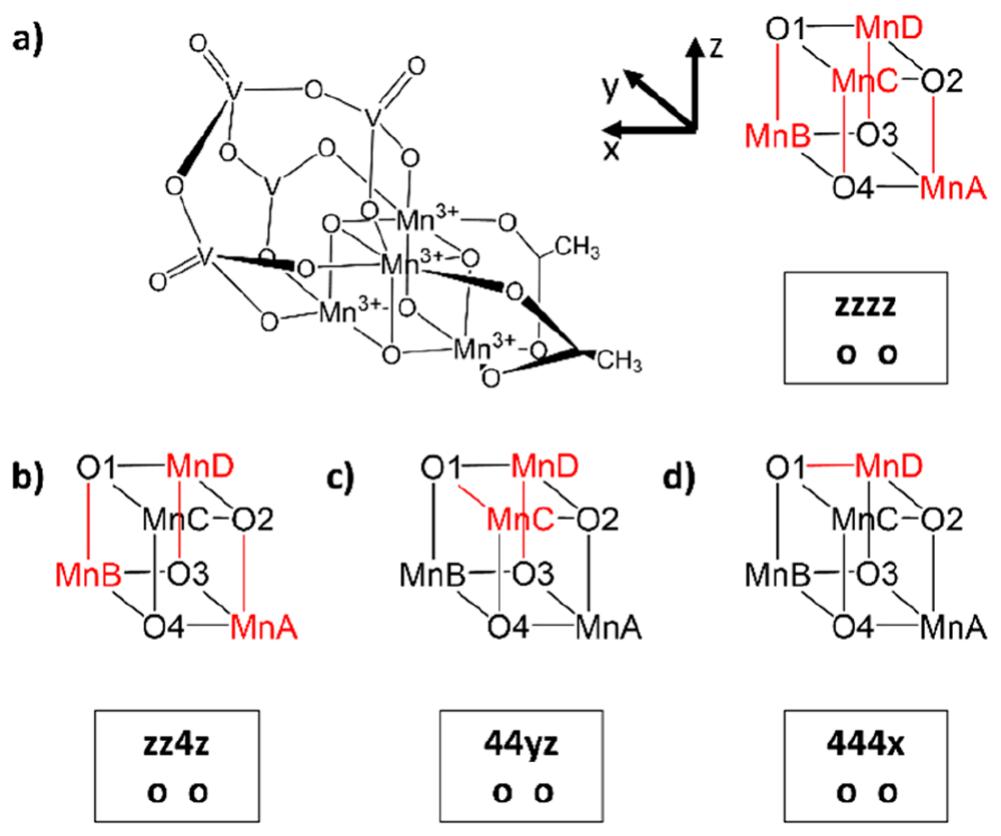
(a) Full ChemDraw structure of **zzzz-o-o**, the most stable isomer of the deactivated species **3333-o-o**, and its abbreviated ChemDraw structure with Mn centers labeled A–D and O atoms labeled 1–4. The Mn^3+^ centers and their respective JT axes are highlighted in red. The accompanying text box contains a descriptor consisting of two parts, the first with letters corresponding to the JT axis orientations at the Mn^3+^ centers ABCD (in this case, **zzzz**) and the second giving ligand configuration (in this case, **o o**, representing open coordination sites at MnB and MnA). (b–d) Abbreviated ChemDraw structures for **zz4z-o-o** (**3334** oxidation state), **44yz-o-o** (**3344**), and **444x-o-o** (**3444**), respectively.

The presence of up to four Mn^3+^ centers across multiple oxidation states of our catalyst, which show distinctive JT bond distortions, necessitates sampling a large number of redox isomers (structures that differ in the assignment of oxidation states to specific Mn centers) and JT isomers (structures that differ in the *x*-, *y*-, or *z*-orientation of their elongated JT bond axes). To differentiate between these isomers, we use a specific nomenclature and abbreviated structural representation that is illustrated in Figure 2. Figure 2a shows the full and abbreviated ChemDraw structures of the most stable JT isomer of the deactivated species **3333-o-o**, which features four Mn^3+^ centers, each with a JT axis pointing to the *z*-direction (highlighted in red), and which will therefore be referred to as **zzzz-o-o**. Further representative examples with other oxidation states are shown in Figures 2b–d. Figure 2b is a **3334-o-o** structure, and because MnA, MnB, and MnD are Mn^3+^ centers with JT axes in the *z*-direction and MnC is an Mn^4+^ center without a JT axis, it is labeled as **zz4z-o-o**. Figure 2c is a **3344-o-o** species, where MnA and MnB are Mn^4+^ centers without JT axes, while MnC and MnD are Mn^3+^ centers with a JT axis in the *y*- and *z*-direction, respectively; it is therefore labeled as **44yz-o-o**. Finally, in Figure 2d, we depict a **3444-o-o** structure, with MnA, MnB, and MnC being Mn^4+^ centers and thus having no JT axes, and MnD being a Mn^3+^ center with a JT axis in the *x*-direction, thus labeled as **444x-o-o**. The **4444** oxidation state of the catalyst does not show redox or JT isomerism.

**Figure 3:**
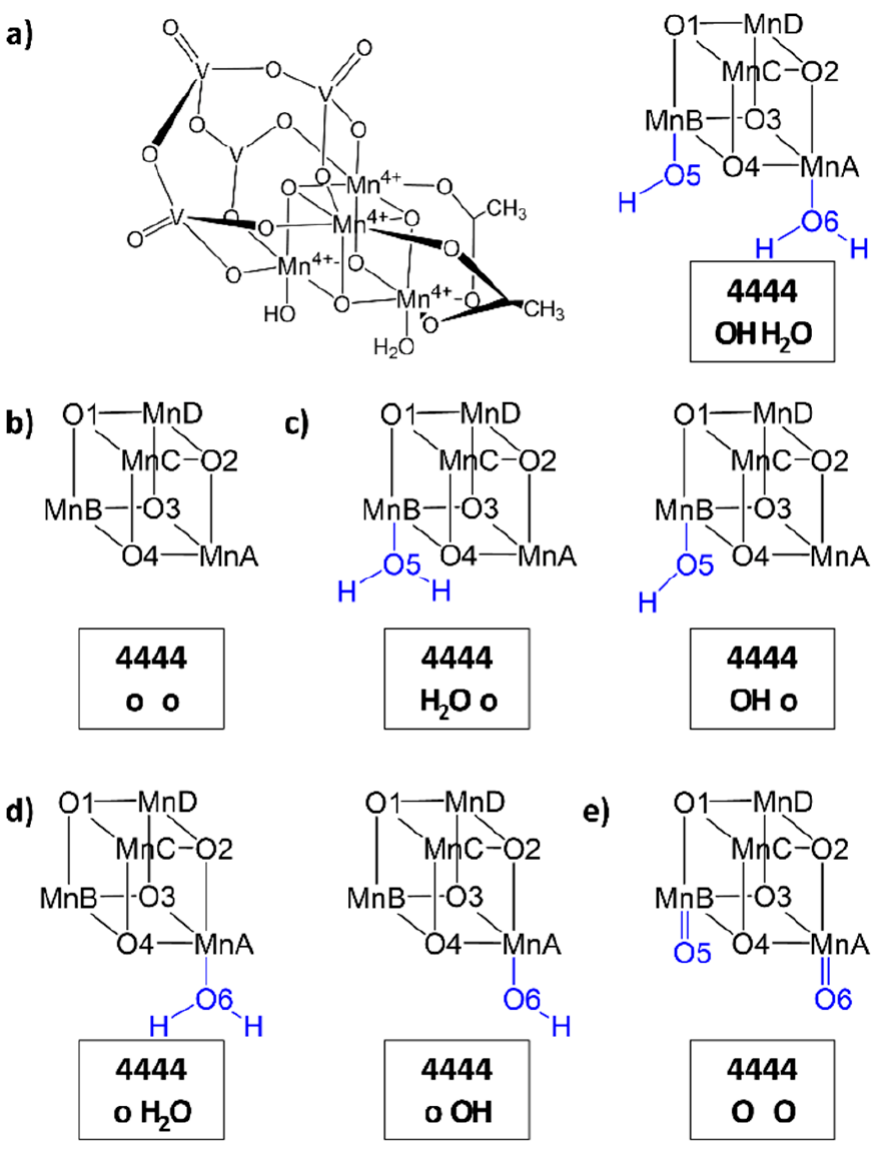
(a) Full ChemDraw structure of the activated species **4444-OH-H**_**2**_**O**, showing the redox state of each Mn center, and its abbreviated ChemDraw structure with Mn centers labeled A-D, O atoms labeled 1–6. Ligands at MnA and MnB are highlighted in blue. The accompanying text box contains a descriptor consisting of two parts; in the first, each number corresponds to the redox state of the Mn^4+^ centers ABCD that have no JT axes (in this case, **4444**) and the ligand configuration (in this case, **OH H**_**2**_**O**, representing the OH ligand at MnB and the H_2_O ligand at MnA). (b–e) Abbreviated ChemDraw structures for **4444-o-o** (panel (b)), **4444-H**_**2**_**O-o** and **4444-OH-o** (panel (c)), **4444-o-H**_**2**_**O** and **4444-o-OH** (panel (d)), and **4444-O-O** (panel (e)).

Furthermore, the catalyst was investigated in a variety of ligand configurations that are exemplified in Figure 3 for the oxidation state **4444**. Figure 3a shows the full and abbreviated ChemDraw structures of **4444-OH-H**_**2**_**O**, a structure with an OH ligand bound to MnB and an H_2_O ligand bound to MnA. Figure 3b displays **4444-o-o**, a structure with an open coordination site at each of the reactive centers MnA and MnB, while Figures 3c and 3d provide two examples each of structures with an open coordination site at MnA and a ligand bound to MnB (**4444-H**_**2**_**O-o** and **4444-OH-o**) and vice versa (**4444-o-H**_**2**_**O** and **4444-o-OH**). Finally, Figure 3e depicts a structure with terminal oxo ligands bound to both MnA and MnB (**4444-O-O**). Other ligand configurations featuring, e.g., acetate ligands bound to MnA and MnB were not investigated here, as this would go beyond the scope of the present study; the reader is instead referred to other work covering the speciation of the catalyst both from an experimental and theoretical point of view.^40,45,46,52,54^

### 2.2 Sampling Procedure

In all investigated ligand configurations, the catalyst exhibits an idealized *C*_*S*_ symmetry, meaning that symmetry-equivalent structures were considered degenerate and therefore not sampled separately. Therefore, all Boltzmann populations are weighted by the degeneracy of the corresponding isomers. Even considering the idealized *C*_*S*_ symmetry of **3344-OAc** derivatives, the complete characterization of a single ligand configuration would have included 136 individual redox and JT isomers, an intractable task considering the number of ligand configurations investigated in this study. Therefore, we made use of the heuristic rules for the relative stability of redox and JT isomers derived by Mai et al.^52^ to predict the most stable isomers for each oxidation state. In the case of the **4444** and **3444** oxidation states, the total number of symmetry-unique isomers is small enough so that all of them could be sampled. However, for the **3344**, **3334**, and **3333** oxidation states, only those isomers predicted by heuristics^52^ to be within less than 12 kcal/mol of the most stable isomer within each oxidation state were targeted for sampling. A detailed analysis of the utility and accuracy of the heuristics used for sampling target selection can be found in section I in the Supporting Information (SI).

Sampling was performed according to a multistep protocol adapted from Mai et al.^52^ Guess geometries for each isomer targeted for sampling were initially obtained from preoptimizations with geometric constraints, wherein a specific JT configuration was enforced by setting the bond length of the corresponding Mn–O bond(s) within the cubane core to a value of 2.30 Å (in some cases, the opposing bond additionally had to be constrained to 2.20 Å; see section II in the SI for details). These guess geometries were then optimized without constraints, resulting in one or more stable isomers for the particular oxidation state and ligand configuration under investigation. As not every targeted isomer corresponded to a stable minimum on the potential energy surface, many of the unconstrained optimizations resulted in virtually identical structures; only the most stable of these for each redox and JT isomer is reported herein.

### 2.3 Computational Details

All single point, optimization, and frequency calculations were performed using the Orca 4.2.1 package,^55,56^ with the zeroth order regular approximation (ZORA),^57−59^ Grimme’s D3 dispersion correction,^60^ and the conductor-like polarized continuum model C-PCM for implicit solvation with surface type vdw_gaussian.^61^ The resolution of identity for Coulomb integrals and numerical chain-of-sphere integration for the Hartree–Fock exchange integrals (RIJCOSX) was used to accelerate the calculations.^62^ Constrained preoptimizations used the looseopt keyword for looser optimization convergence thresholds, with the unrestricted BP86 functional^63,64^ and the ZORA-SVP basis set with def2/J general auxiliary basis set.^65^ For simplicity, the C-PCM parameters of acetonitrile were used. Final unconstrained optimizations and numerical frequency calculations of isomers made use of the unrestricted B3LYP functional,^66,67^ with the double-ζ ZORA-def2-SVP basis set and SARC/J decontracted auxiliary basis set.^65^ To simulate the ACN:H_2_O 9:1 (v/v) solvent mixture employed in experiments,^45,46^ the C-PCM parameters for acetonitrile were combined with a custom epsilon value of 41.589 (9:1 weighted average of the epsilon values of ACN and H_2_O).^41^ Finally, single point electronic energies were refined using the larger triple-ζ ZORA-def2-TZVP basis set^65^ with otherwise identical parameters to the final optimization protocol. The JT configuration of optimized structures was determined by comparing the lengths of Mn–O bonds within the cubane core, with the longest bond determining the *x*-, *y*-, or *z*-orientation of the JT axis on each Mn^3+^ center. All obtained structures were evaluated according to their numerically calculated vibrational frequencies; those showing negative frequencies were excluded from the results discussed herein. In all our calculations, an all-atom model of the complex using the high-spin configuration was employed, as is common in the literature.^40,41,52,54,68−70^ Oxidation states are reported based on the Mulliken spin populations computed for individual atoms.

Final reported Gibbs energies are based on the refined electronic energies calculated using the ZORA-def2-TZVP basis set in combination with thermochemical corrections from the final optimizations and frequency calculations carried out using the ZORA-def2-SVP basis set. To account for energy differences between different ligand configurations, reference energies of the isolated ligands were calculated at the same final level of theory. Free energies in solution have been corrected for concentration effects using the package “GoodVibes”,^71^ setting the concentrations of all catalyst intermediates to a standard reference value of 1 M and the concentration of the 10% water in solution to 5.53 M.^72^ Furthermore, to account for differing protonation states between structures, an energy correction term was calculated using the approach of Van Voorhis and co-workers, wherein the standard free energy of a proton in solution is added for each proton abstracted from the cluster.^8^ As in our previous work,^41^ we used a 9:1 weighted average of the standard free energy of a proton in acetonitrile (11.0622 eV) and of a proton in water (11.5305 eV)^73^ to approximate the standard free energy of a proton in the ACN:H_2_O 9:1 (v/v) solution mixture used in the experiment, giving 11.1090 eV.

In order to study possible instances of catalyst degradation, Climbing Image-Nudged Elastic Band (CI-NEB)^74^ calculations, as implemented in ORCA 5.0.3,^55,56^ were performed on several examples of intermediates showing various structural defects. Here, the unrestricted B3LYP functional^66,67^ was used with the ZORA-def2-SVP basis set,^65^ D3 dispersion correction,^60^ and C-PCM (acetonitrile, epsilon = 41.589) with surface type vdw_gaussian.^61^ Barrier heights for reactions studied using CI-NEB reported herein are calculated based on the electronic energy difference between the NEB climbing image and the reactant species, computed using ZORA-def2-SVP, and should therefore be taken as approximate, most likely upper bounds.

## 3 Results

**Figure 4:**
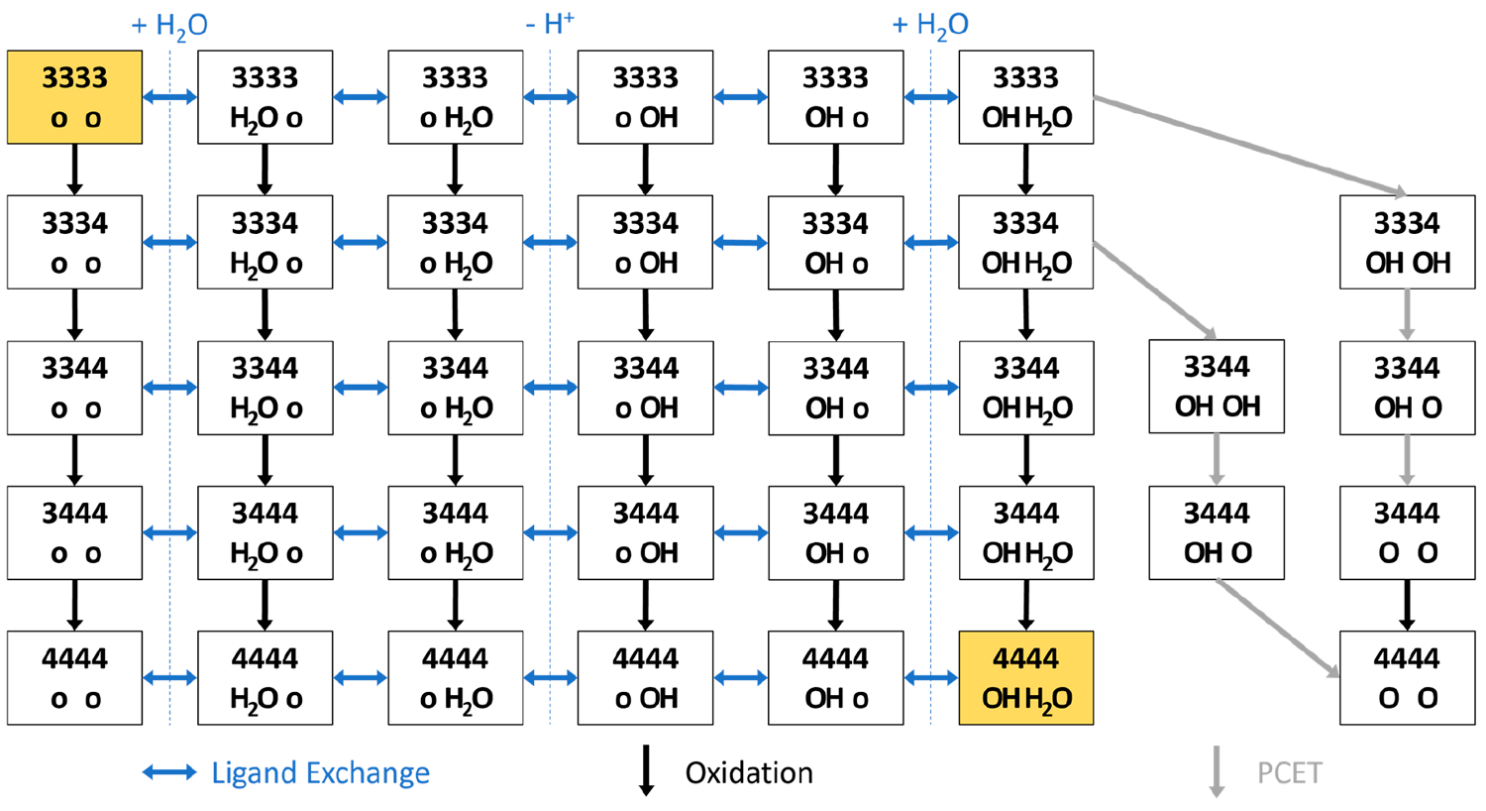
Scheme of all investigated oxidation pathways and their intermediates. Ligand exchange reactions are indicated by blue arrows, oxidation steps by black arrows, and proton-coupled electron transfer (PCET) steps by gray arrows. The starting **3333-o-o** and final **4444-OH-H**_**2**_**O** structures (derived from prior work^40,41^) are highlighted in orange.

A total of 558 individual isomers were sampled across all oxidation states and ligand configurations, resulting in 159 stable minima. All investigated combinations of oxidation states and ligand configurations are shown in Figure 4. In the following, obtained results will be summarized for each ligand configuration, focusing on the most stable minima for each oxidation state—i.e., listing all structures with a Boltzmann population of at least 5% at thermal equilibrium (*T* = 298.15 K) for that ligand configuration and oxidation state. A full list of all optimized structures can be found in Tables S2–S9 in the SI.

Analysis of the bond lengths between each metal center and its coordinating atoms revealed several structures showing varying degrees of ligand dissociation; these structures are marked with an asterisk (*), and corresponding large interatomic distances are noted explicitly. To study the processes leading to the formation of such partially dissociated intermediates, NEB calculations were performed between representative examples of these intermediates and nondissociated structures of identical oxidation state and comparable ligand configuration. The energy values given for intermediates and their various isomers are relative Gibbs energies calculated at the B3LYP-D3/ZORA-def2-TZVP//B3LYP-D3/ZORA-def2-SVP level of theory, while barrier heights are the relative electronic energy between the reactant and the climbing image of a CI-NEB simulation carried out only at the B3LYP-D3/ZORA-def2-SVP level of theory.

We begin at the lowest oxidation state with the ligand configuration featuring two open coordination sites, **3333-o-o**, which is the starting point of catalyst regeneration. The most stable isomer is zzzz-o-o* (0.0 eV relative Gibbs energy, 68% Boltzmann population at *T* = 298.15 K), which is used as a reference structure throughout this work. It features two slightly longer-than-average bond lengths within the cubane core caused by strong JT effects (*r*(MnC–O4) = 2.579 Å, *r*(MnD–O3) = 2.549 Å). Two other populated JT isomers were found: zzyz-o-o* (0.04 eV, 26%, *r*(MnD–O3) = 2.776 Å) and **zzzz-o-o** (0.06 eV, 5%). A NEB calculation between **zzzz-o-o** and zzzz-o-o* revealed a low kinetic barrier between them amounting to 0.25 eV or 5.7 kcal/mol. Oxidation to the **3334** state yields a mixed population consisting of zz4z-o-o* (3.95 eV, 90%, *r*(MnD–O3 = 2.519 Å) and **zz4x-o-o** (4.01 eV, 10%). A corresponding NEB calculation between **zz4x-o-o** and zz4z-o-o* showed that the elongation within the cubane core of the bond between MnD and O3 is effectively barrierless, with an activation energy of only 0.04 eV or 0.9 kcal/mol. Further oxidation states are dominated by single isomers: **zz44-o-o** (8.71 eV, 100%) for the **3344** oxidation state, **4z44-o-o** (14.49 eV, 100%) for the **3444** oxidation state, and finally **4444-o-o** (21.19 eV, 100%) for the **4444** oxidation state.

Moving on to the ligand configuration featuring a H_2_O bound at MnB and an open coordination site at MnA, the most stable structures found at the lowest oxidation state (corresponding to **3333-H**_**2**_**O-o** in Figure 4) are **xzxx-H**_**2**_O-o* (0.05 eV, 57%, *r*(MnC–O2) = 2.548 Å, *r*(MnB–O5) = 3.526 Å, *r*(MnD–OAc) = 4.450 Å) and **zzzx-H**_**2**_O-o* (0.05 eV, 43%, *r*(MnC–O4) = 2.884 Å, *r*(MnB–O5) = 3.851 Å). These structures, along with all but one of the others found for this ligand configuration and oxidation state, feature dissociated ligands—the sole exception being **zxyx-H**_**2**_**O-o** (0.50 eV), which is however unpopulated at thermal equilibrium. At the **3334** oxidation state, two stable, populated intermediates could be identified: **z4yx-H**_**2**_**O-o** (3.94 eV, 53%) and **zz4x-H**_**2**_**O-o** (3.96 eV, 47%). The next oxidation leads to **z4y4-H**_**2**_**O-o** (8.51 eV, 100%); further oxidation yields a mixture of **z444-H**_**2**_**O-o** (13.95 eV, 79%) and **444x-H**_**2**_**O-o** (14.00 eV, 21%); and finally **4444-H**_**2**_**O-o** (20.22 eV, 100%) is obtained.

For the ligand configuration with an open coordination site at MnB and H_2_O bound at MnA, the most stable isomer corresponding to **3333-o-H**_**2**_**O** is **xzzx-o-H**_**2**_O* (−0.37 eV, 100%, *r*(MnC–O4) = 3.014 Å), featuring an unusual combination of opened cubane core and protonated O4. A NEB calculation between the unpopulated **xzxx-o-H**_**2**_**O** (0.43 eV) and **xzzx-o-H**_**2**_O* reveals a very low barrier of only 0.17 eV or 3.8 kcal/mol for conversion to the O4-protonated species. The remaining oxidation states are dominated by only one populated species. Accordingly, starting at the **3334** oxidation state with **zzy4-o-H**_**2**_**O** (3.92 eV, 98%), oxidation to the **3344** oxidation state yields **4z4x-o-H**_**2**_**O** (8.38 eV, 100%), further oxidation to the **3444** oxidation state yields **4z44-o-H**_**2**_**O** (13.57 eV, 100%), and finally oxidation to **4444** yields **4444-o-H**_**2**_**O** (20,11 eV, 100%).

Next, we examine the most stable intermediates for the ligand configuration featuring an open coordination site at MnB and OH bound at MnA. For the lowest oxidation state, the most stable isomer of **3333-o-OH** is **xzyz-o-OH** (1.86 eV, 95%). Oxidation to the **3334** oxidation state yields a mixed population of **yz4x-o-OH** (5.25 eV, 63%) and **yzy4-o-OH** (5.26 eV, 37%). Further oxidation to the **3344** oxidation state gives **4zy4-o-OH** (9.28 eV, 99%), then oxidation to the **3444** oxidation state gives **4z44-o-OH** (14.04 eV, 100%), and finally oxidation to the **4444** oxidation state gives **4444-o-OH** (20.23 eV, 100%).

All isomers found for **3333-OH-o**, the lowest oxidation state for the ligand configuration featuring OH bound at MnB and an open coordination site at MnA, have at least one partially dissociated ligand. The most stable isomers are xxxx–OH-o* (1.88 eV, 71%, *r*(MnB–O3) = 2.531 Å, *r*(MnD–OAc) = 4.810 Å) with an acetate ligand dissociated from one of its metal centers resulting in an open coordination site at MnD and yyyx-OH-o* (1.90 eV, 29%, *r*(MnB–O4) = 2.807 Å, *r*(MnC–OAc) = 6.651 Å) with a corresponding open coordination site at MnC. A NEB calculation was performed between the unpopulated isomers zzyx-OH-o* (2.84 eV, *r*(MnC–OAc) = 2.564 Å, *r*(MnD–OAc) = 2.556 Å) and zxyx-OH-o* (2.03 eV, *r*(MnD–OAc) = 4.157 Å) to investigate the transition between a strongly distorted and a fully dissociated Mn–OAc bond, finding a very low barrier of only 0.08 eV or 1.9 kcal/mol. Moving on to the **3334** oxidation state, only one populated isomer, **z4yz-OH-o** (5.44 eV, 99%), could be optimized. Oxidation to the **3344** oxidation state yields **z44x-OH-o** (9.50 eV, 100%); further oxidation to **3334** then results in **z444-OH-o** (14.45 eV, 100%), and finally oxidation to **4444** gives **4444-OH-o** (20.35 eV, 100%).

The final ligand configuration investigated as part of the regeneration half of the iWNA cycle from **3333-o-o** to **4444-OH-H**_**2**_**O** features an OH and an H_2_O ligand. One should note that the ligands at the two binding sites MnA and MnB are essentially interchangeable due to their hydrogen-bonded nature. However, thermodynamically it is more favorable to have H_2_O bound to MnB for all oxidation states except the **4444** oxidation state. At the **3333** oxidation state, the most stable structures are **yzzx-H**_**2**_O-OH* (1.91 eV, 93%, *r*(MnB–O5) = 3.166 Å) with a dissociated H_2_O ligand and **xxyx-H**_**2**_**O–OH** (1.98 eV, 5%). To obtain an approximate barrier height for the dissociation of an H_2_O ligand, a NEB calculation was performed between **xxyx-H**_**2**_**O-OH** and **yzzx-H**_**2**_O-OH*, resulting in a low barrier of 0.23 eV or 5.4 kcal/mol. Oxidizing to the **3334** oxidation state results in a mixed population of **yz4x-H**_**2**_**O-OH** (5.13 eV, 84%) and **xz4x-H**_**2**_**O-OH** (5,18 eV, 13%). Further oxidation to the **3344** oxidation state gives both **4z4x-H**_**2**_**O-OH** (9.05 eV, 91%) and **44yx-H**_**2**_**O-OH** (9.10 eV, 6%), while further oxidation to the **3444** oxidation state yields a mixture of **4z44-H**_**2**_**O-OH** (13.69 eV, 58%) and **444x-H**_**2**_**O-OH** (13.71 eV, 42%). The final oxidation step leads to **4444-OH-H**_**2**_**O** (19.26 eV, 70%) and **4444-H**_**2**_**O-OH** (19.28 eV, 30%), illustrating the typically small energy difference between the two protonation states.

We turn now to intermediates from a possible alternative iDC-type water oxidation cycle (gray in Figure 1b). The two iDC regeneration pathways we investigated (Figure 4) start from **3333-o-o** and proceed through the binding of water and a combination of PCET steps and one-electron oxidations all the way to **4444-O-O**, which contains two terminal oxo groups, ready to form an O–O bond by direct coupling. The initial intermediates of the first pathway feature an H_2_O ligand bound to MnB and an OH ligand bound to MnA, resulting in two JT isomers: **yzzx-H**_**2**_O-OH* (1.91 eV, 93%, *r*(MnB-O5) = 3.166 Å) and **xxyx-H**_**2**_**O-OH** (1.98 eV, 5%). The first PCET step also results in a mixture of isomers, **yy4x-HO-HO** (7.09 eV, 54%), with O6 as the H-bond donor and O5 as the acceptor, and **xx4x-OH-OH** (7.10 eV, 40%) with O5 as the H-bond donor. Also at this step, a NEB calculation was performed between the unpopulated isomers **4xyx-OH-OH** (7.31 eV) and 4xxx-OH-OH* (7.40 eV, *r*(MnD–OAc = 2.548 Å)), both with O5 as the H-bond donor and O6 as the H-bond acceptor. The barrier for the transition from the JT-distorted MnD–OAc bond of **4xyx-OH-OH** to the very strongly JT-distorted MnD–OAc bond of 4xxx-OH-OH* was found to be very low at 0.17 eV or 4.0 kcal/mol. The second PCET step gives a mixed population of **4y4x-OH-O** (13.30 eV, 34%), **4x4x-OH-O** (13.30 eV, 27%), **x44x-O-OH** (13.31 eV, 18%), and **y44x-O-OH** (13.32 eV, 14%). The final PCET step yields **444x-O-O** (19.66 eV, 100%), which can be oxidized to **4444-O-O** (23.58 eV, 100%). Some spin delocalization across the Mn–O bonds can be observed for the deprotonated terminal oxo groups (see Table S8 in the SI).

Finally, the initial intermediates of the second iDC regeneration pathway investigated also feature an H_2_O and an OH ligand: **yzzx-H**_**2**_O-OH* (1.91 eV, 93%, *r*(MnB–O5) = 3.166 Å) and **xxyx-H**_**2**_**O-OH** (1.98 eV, 5%), then **yz4x-H**_**2**_**O-OH** (5.13 eV, 84%) and **xz4x-H**_**2**_**O-OH** (5,18 eV, 13%). The first PCET step yields a mixed population of **y44x-HO-HO** (10.64 eV, 30%) with O6 being the H-bond donor and O5 the acceptor, **x44x-HO-HO** (10.65 eV, 24%) with the same H-bonding configuration, **y44x-OH-OH** (10.66 eV, 16%) with the inverted configuration of O5 being the H-bond donor and O6 the acceptor, **x44x-OH-OH** (10.66 eV, 11%) with the same inverted configuration, and **4x4x-HO-HO** (10.67 eV, 11%) once again having O6 as the donor. A further PCET step gives a mixture of **444x-OH-O** (16.90 eV, 87%) and **444x-O-OH** (16.95 eV, 13%). The final PCET step results in the same final product as the previous pathway, **4444-O-O** (23.58 eV, 100%). Again, some spin delocalization across the Mn–O bonds can be observed for the deprotonated terminal oxo groups (see Table S9 in the SI).

## 4 Discussion

### 4.1 Structural Analysis

**Figure 5:**
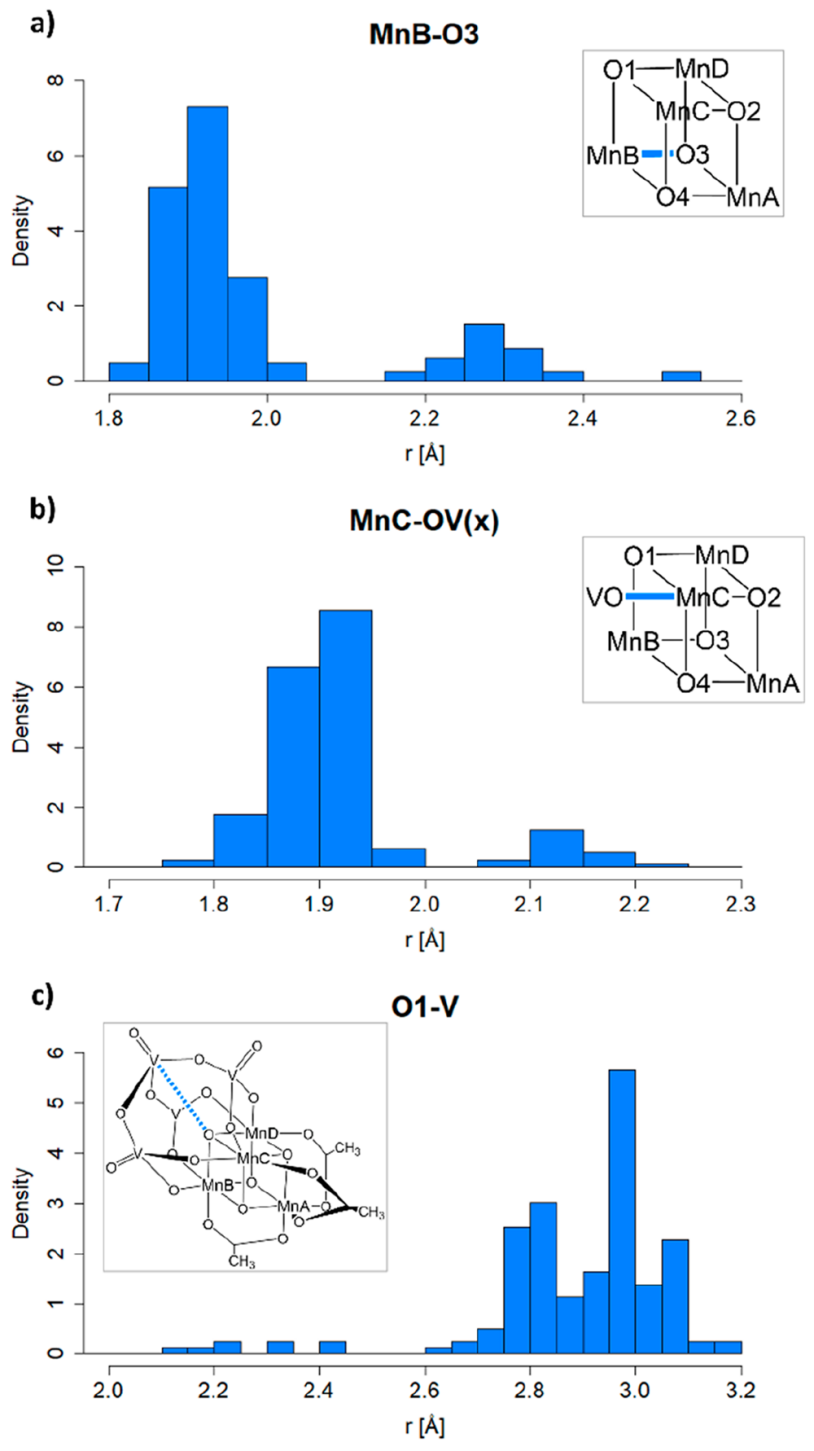
Interatomic distance distributions (in Å) for selected atom pairs highlighted in blue in the insets. (a) *r*(MnB–O3), showing two peaks: one at ∼2.3 Å for JT-distorted bonds and one around 1.9 Å for bonds not lengthened by JT effects. An outlier above 2.5 Å represents possibly dissociated structures. (b) *r*(MnC–OV), where OV is the nearest O atom of the vanadate ligand. The presence of a second peak of ∼2.1 Å indicates that bonds to the vanadate ligand are affected by JT distortions. (c) *r*(O1–V), where V is the nearest V atom of the vanadate ligand. Outliers below 2.6 Å show that an attractive interaction between these two atoms can pull them up to 0.4 Å closer together.

Analyzing the distributions of the interatomic distances between the Mn centers and each of their coordinating atoms reveals a common pattern (see section III in the SI), shown exemplarily in Figure 5a for *r*(MnB–O3). A peak at ∼1.9 Å represents both the undistorted bonds found at Mn^4+^ centers as well as the slightly shorter bonds not corresponding to the main JT axis at Mn^3+^ centers. A second peak at ∼2.3 Å represents bonds lengthened by JT distortions. Outliers above 2.5 Å correspond to structures featuring very strongly distorted or even fully dissociated bonds, which will be discussed in detail below.

This pattern of interatomic distance distributions marked by two main peaks is found essentially in all Mn centers and coordinating atoms, showing that JT distortions are possible along all bond axes, including those to the vanadate ligand (see Figure 5b). Overall, we predict 78 structures bearing at least one JT axis oriented toward the vanadate ligand, 18 of which have a Boltzmann population of >5% at thermal equilibrium, marking them as being among the most stable isomers. While the majority of these structures belong to the **3333** and **3334** oxidation states, 17 such structures were found at the **3344** and 1 structure was found even at the **3444** oxidation state. This is a surprising result because it diverges somewhat from the assumption that the seemingly chemically inert vanadate ligand was unlikely to participate in the formation of JT axes.^52^

Rather, it appears that JT distortions in bonds to the vanadate ligand are possible, although they result in bonds that are more stable than is the case for other ligands (bonds to the vanadate lengthened by JT distortions are on average 0.1–0.2 Å shorter than JT-lengthened bonds to other ligands), and no instances of bond dissociation were observed for the vanadate ligand.

Interestingly, we obtained a number of structures featuring an interatomic distance smaller than 2.5 Å between O1 and one of the vanadium atoms (Figure 5c), indicating a certain degree of interaction between the two atoms. As previously noted by Mai et al., the experimental X-ray structure obtained at the **3344** oxidation state features three elongated Mn–O1 bonds, which were originally rationalized as being caused by attractive electrostatic interactions between O1 and the vanadate ligand,^45^ but are more likely a result of dynamic disorder in the crystal structure between different JT isomers separated by low kinetic barriers.^52^ Now, however, we find structures in the **3333** and **3334** oxidation state, all of which have three JT axes pointed at O1 and significantly lower than average distances between O1 and the vanadate ligand. This implies that the hypothesized attractive interaction of this pairing could be observed, but only at the lowest oxidation states and with the cooperation of three JT axes pushing O1 toward the vanadate ligand.

### 4.2 Catalyst Degradation

The appearance of numerous outliers in the interatomic distance analysis prompted us to investigate in depth structures featuring very strongly distorted or even fully dissociated bonds between Mn centers and their coordinating atoms. We discovered two types of potential catalyst degradation processes: (i) cubane opening, where intracubane Mn–O bonds are extended up to 3.0 Å, in one case followed by protonation of O4 by a neighboring H_2_O ligand; and (ii) ligand dissociation, where Mn–OH_2_ distances up to 3.9 Å and Mn–OAc distances up to 6.7 Å can be observed. Structures bearing one or even several of these distortions were found both in the **3334** oxidation state, where they appear in a small number of mostly unpopulated isomers, and in the **3333** oxidation state, where they are far more common and often among the most stable isomers obtained. In all these structures, Mn-ligand bond distortions and dissociations are consistent with the JT axes of their respective Mn^3+^ centers. Therefore, it is reasonable to assume that here, as in other parts of the catalytic cycles,^40,41^ JT effects lower barriers for bond breakage and formation at Mn^3+^ centers, in this case potentially facilitating catalyst degradation through cubane opening and ligand dissociation.

**Figure 6:**
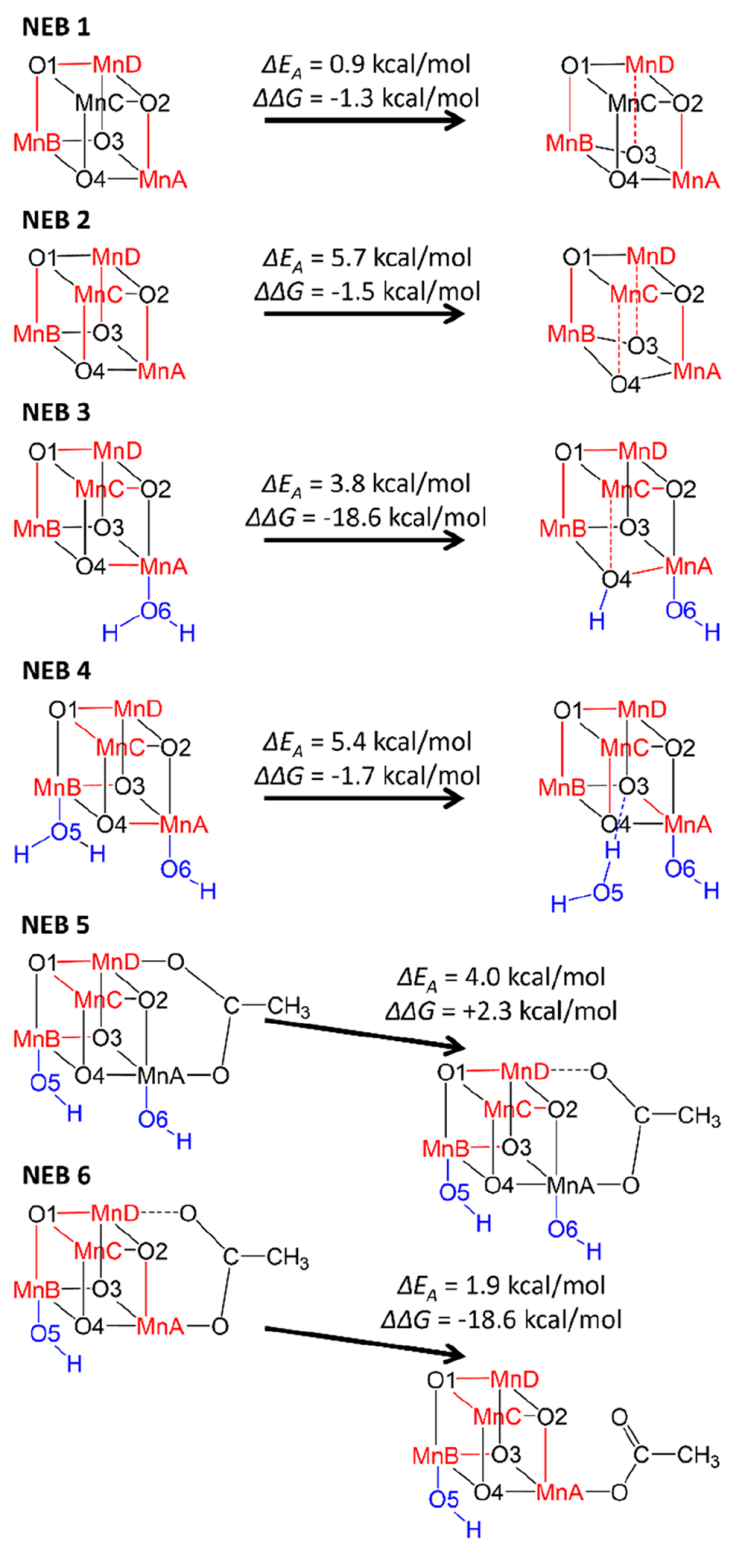
CI-NEB calculations carried out between relatively intact isomers and examples featuring at least one form of structural distortion. Mn^3+^ centers and their JT axes are marked in red; ligands at MnA and MnB are highlighted in blue. Activation energies Δ*E*_A_ (kcal/mol) correspond to the electronic energy of the climbing image relative to the reactant, computed at the B3LYP-D3/ZORA-def2-SVP level of theory. Gibbs energy differences ΔΔ*G* (in kcal/mol) are calculated at the B3LYP-D3/ZORA-def2-TZVP//B3LYP-D3/ZORA-def2-SVP level of theory.

To better understand the kinetics of these degradation processes, NEB calculations were performed between representative examples of structures bearing strongly distorted or dissociated bonds and nondissociated isomers of identical oxidation state and comparable ligand configuration. In this way, approximate barrier heights for different types of cubane opening and ligand dissociation processes could be obtained. Six such calculations were performed (see Figure 6 and section IV in the SI); in some cases (NEB 1, NEB 2), the minimum energy pathway obtained from the NEB calculation fluctuates between several JT isomers due to the near-degeneracy of many isomers at the **3333** oxidation state. However, the emergence of JT distortions consistent with the studied degradation process was observed before bond cleavage in two simulations (NEB 3, NEB 4), while in the remaining calculations, such JT distortions were already present in the reactant structure.

The barriers of all degradation processes investigated using NEB simulations were found to be small (<0.25 eV or 5.8 kcal/mol), pointing to the high reactivity of the catalyst at the **3333** and **3334** oxidation states. The catalyst’s propensity for quickly dissociating ligands is enabled by the presence of JT effects at Mn^3+^ centers, which can extend Mn-ligand bonds and thereby lower reaction barriers for bond dissociation. Based on these results, it appears that all of these extremely fast reactions are potentially reversible, depending on the ΔΔ*G* between reactant and product. For example, the products of NEB 1 and NEB 2 featuring extended intracubane Mn–O bonds, zzzz-o-o* and zz4z-o-o*, are in equilibrium with their “intact” reactants. In these two cases, cubane opening does not seem to interfere with oxidative regeneration of the catalyst and is simply a form of a particularly strong JT effect. Similarly, the H_2_O and acetate ligand dissociations studied in NEB 4 and NEB 5, respectively, appear to be reversible, showing that (partial) ligand dissociation does not necessarily impede regeneration. This is plausible in the case of H_2_O, which is abundant in the reaction mixture under experimental conditions.^45,46^ However, the dissociation of acetate from the complex bears further investigation (see below).

While NEB 5 shows that extreme distortion of an Mn–OAc bond (*r*(MnD–OAc) = 2.548 Å in the product) is reversible, this is not the case in NEB 6. In the latter, the bidentate acetate ligand is fully dissociated from MnD, with the remainder being bound as a monodentate ligand at MnA and leaving behind an additional open coordination site. This far more dramatic reaction is associated with a large negative ΔΔ*G*, pushing the system irreversibly toward the degraded product. NEB 3 falls into the same category; there, a combination of cubane opening and protonation by an H_2_O ligand at O4 irreversibly results in the formation of a unique degradation product, **xzzx-o-H**_**2**_O*, that is fully 0.37 eV lower in energy than the next most stable isomer, zzzz-o-o*. It would seem that this process could cause potentially irreversible damage to the catalyst, as the breaking of a μ-oxo bridge in the cubane core might interfere with the facile transfer of electrons between Mn centers, leading to a reduction in catalytic activity as this degradation product accumulates and possibly disintegrates even further. However, the irreversible protonation at O4 first requires H_2_O to be bound as a ligand, which is thermodynamically quite unfavorable at the **3333** oxidation state: The most stable isomer that includes an H_2_O ligand bound to either active site is the thermally unpopulated xzyz-H2O-o* (0.29 eV, *r*(MnD–O3) = 2.619 Å); this isomer, in turn, is 0.29 eV less stable than the reference structure without H_2_O ligands, zzzz-o-o*. Alternatively, O4 could be protonated by a solvent water molecule to form **xzzx-o-H**_**2**_O*. As the catalyst has been shown in experiments to catalyze over 12 000 turnovers,^46^ we can safely assume that deactivation by solvent-based protonation of O4 must be associated with a substantial kinetic barrier. The further investigation of this intriguing process would require the inclusion of explicit solvent dynamics and is therefore beyond the scope of this work.

### 4.3 Catalyst Regeneration (iWNA Cycle)

**Figure 7:**
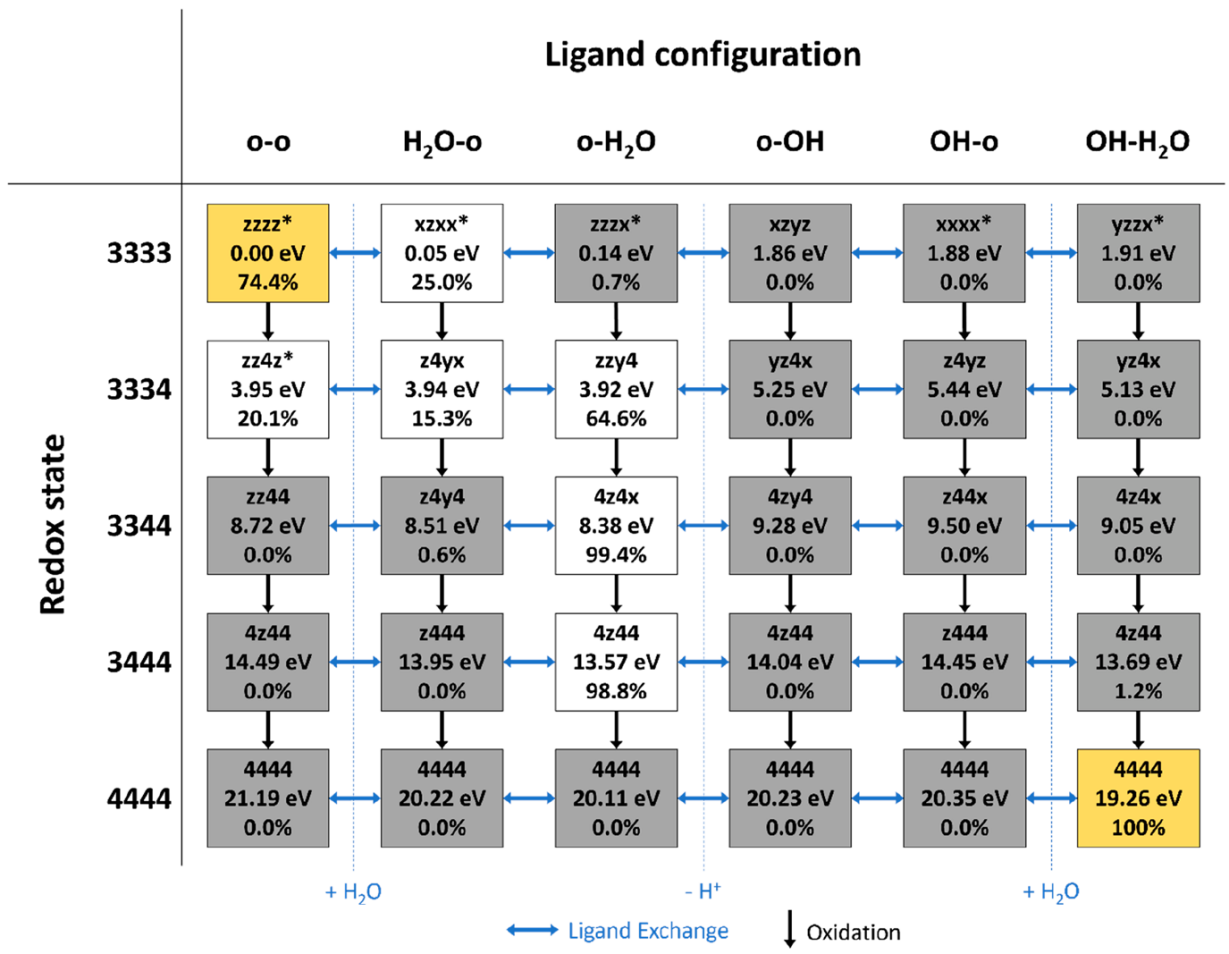
Most stable intermediates for each oxidation state (left) and ligand configuration (top), together comprising the most thermodynamically favorable regeneration pathways within the iWNA cycle. For each structure, the text box specifies JT isomer, relative Gibbs free energy (in eV), and relative Boltzmann population at 298.15 K within the given oxidation state. The starting **3333-o-o** and final **4444-OH-H**_**2**_**O** structures are highlighted in orange, intermediates with a relative population smaller than 5.0% are shaded in gray. Ligand exchange reactions are indicated by blue arrows, and oxidation steps are indicated by black arrows.

Leaving aside possible degradation products (in particular, **xzzx-o-H**_**2**_O*), we now turn to the question of how **4444-OH-H**_**2**_**O** is regenerated from **3333-o-o** within the iWNA catalytic cycle of **3344-OAc**. As noted above, this regeneration reaction formally involves oxidation as well as ligand binding steps, making the detailed characterization of a single preferred reaction pathway extremely difficult. The binding of new water ligands to **3344-OAc** could occur at any oxidation state and has been shown to be subject to reaction barriers of widely differing heights in the context of catalyst activation.^40^ Therefore, we choose to focus here solely on the thermodynamics of catalyst regeneration by identifying the most stable intermediates for each oxidation state and ligand configuration, shown in Figure 7.

To simplify the discussion, let us imagine that in place of the many JT and redox isomers that we have sampled, only the most stable isomer for each combination of redox state and ligand configuration were thermally populated. This would allow us to more easily compare intermediates featuring different ligand configurations at the same oxidation state, enabling us to estimate which of these intermediates could be populated at thermal equilibrium (orange and white boxes in Figure 7). Although unpopulated intermediates (gray boxes in Figure 7) may also play a role in catalyst regeneration depending on the reaction conditions (e.g., applied overpotential in electrocatalysis), a regeneration pathway featuring only the most stable intermediates would result in the lowest thermodynamic overpotential.^7,75,76^

Starting at the **3333** oxidation state, an isomer of the **o-o** ligand configuration (zzzz-o-o*, 0.00 eV, 74.4%) is in thermodynamic equilibrium with a structure bearing dissociated H_2_O and acetate ligands (**xzxx-H**_**2**_O-o*, 0.05 eV, 25.0%). It appears that having open coordination sites at both MnA and MnB is the most stable ligand configuration at the **3333** oxidation state. Oxidation to the **3334** state results in an equilibrium between structures with one open coordination site as well as one H_2_O ligand, **zzy4-o-H**_**2**_**O** (3.92 eV, 64.6%) and **z4yx-H**_**2**_**O-o** (3.94 eV, 15.3%), and a structure with two open coordination sites, zz4z-o-o* (3.95 eV, 20.1%). Oxidizing to the **3344** oxidation state, the **o-H**_**2**_**O** configuration with an open coordination site at MnB and H_2_O bound to MnA becomes the most stable by far (**4z4x-o-H**_**2**_**O**, 8.38 eV, 99.4%). This behavior is also found at the **3444** oxidation state, where **4z44-o-H**_**2**_**O** (13.57 eV, 98.8%) is the only significantly populated intermediate. At the highest oxidation state **4444**, the expected final product **4444-OH-H**_**2**_**O** (19.26 eV, 100%) is also the most stable structure.

Table 1 shows the oxidation potentials of the oxidation steps connecting the thermally populated intermediates in Figure 7 (ranges are given where several intermediates are populated). We note that the potential of the last oxidation step is the highest of the overall reaction: 1.41 V versus normal hydrogen electrode (NHE) for **4z44-o-H**_**2**_**O** to **4444-OH-H**_**2**_**O**. This value is just below the redox potential of the **3444-OH-H**_**2**_**O** to **4444-OH-H**_**2**_**O** oxidation observed experimentally during catalyst activation, 1.47 eV vs NHE,^40^ but slightly higher than the potential of the same oxidation step in a comparable OEC mimic, 1.3 V vs NHE.^77^ These results indicate that oxidative regeneration of the **3344-OAc** catalyst is energetically comparable to its activation as well as to parallel processes in comparable structures.

**Table 1:** Oxidation Potentials versus Normal Hydrogen Electrode *E*_NHE_ for Oxidation Steps between Thermally Populated Intermediates Shown in Figure 7

oxidation step	*E*_NHE_^a^ [V]
**3333** → **3334**	[−0.33; −0.41]
**3334** → **3344**	[0.15; 0.18]
**3344** → **3444**	0.91
**3444** → **4444**	1.41

a Potential ranges are given where several populated intermediates are present.

### 4.4 Alternative Regeneration Pathways (iDC Cycle)

Finally, we discuss two alternative regeneration pathways enabling an iDC-type water oxidation cycle. We hypothesized that O_2_ evolution according to such a mechanism could best be enabled by first binding water ligands at a low redox state (**3333** or **3334**), followed by combined deprotonation and metal-centered oxidation through a series of PCET steps, leading to a highly oxidized catalyst with terminal oxo ligands (**4444-O-O**). For the pathway starting from **3333-H**_**2**_**O-OH**, already the first PCET step leads to a structure (**yy4x-HO-HO**) that is 1.65 eV higher in energy than the next most stable isomer belonging to the iWNA cycle, **z4yz-OH-o**. A similar result is obtained when starting instead from **3334-H**_**2**_**O-OH**, with the first PCET step leading to **y44x-HO-HO**, which is 1.14 eV less stable than the next most stable isomer from the iWNA cycle, **z44x-OH-o**. All further intermediates from these two iDC regeneration pathways are also far less stable than iWNA regeneration intermediates at the same oxidation state. We therefore conclude that these particular iDC-type regeneration pathways play no significant role in the reactivity of our catalyst, although a different type of DC water oxidation cycle may, of course, yet be discovered.

## 5 Conclusion

We determined the most favorable regeneration mechanism of the biomimetic polyoxometalate water oxidation catalyst [Mn^3+^_2_Mn^4+^_2_V_4_O_17_(OAc)_3_]^3–^ (**3344-OAc**), thereby completing the iWNA-type catalytic cycle. Starting from its least oxidized **3333-o-o** form with two open coordination sides, the first H_2_O ligand is able to bind at the **3334** oxidation state. This oxidation state shows the greatest diversity of populated ligand configurations. Afterward, the reaction converges to a single pathway leading via **3344-o-H**_**2**_**O** and **3444-o-H**_**2**_**O** back to the activated species **4444-OH-H**_**2**_**O** as the final product. The second H_2_O ligand is bound and deprotonated together with the final oxidation step. The catalyst’s ability to access a variety of JT as well as redox isomers and ligand configurations is key to achieving a highly efficient catalytic cycle, starting with initial activation of the precatalyst **3344-OAc** to **4444-OH-H**_**2**_**O**,^40^ then O_2_ evolution leaving behind the deactivated **3333-o-o**,^41^ and finally regeneration of **4444-OH-H**_**2**_**O**.

Additionally, we investigated the feasibility of an alternative iDC-type water oxidation cycle, initiated by binding water ligands to **3333-o-o** followed by a series of PCET steps to attain a highly oxidized species with two cofacial terminal oxo ligands (**4444-O-O**). However, this alternative regeneration pathway did not turn out to be thermodynamically favorable.

Since JT effects play a major role in determining the reactivity and stability of this polyoxometalate water oxidation catalyst, a tremendous in silico sampling effort was indispensable to characterize the involved structures, resulting in 159 individual stable minima found. This large-scale theoretical investigation not only offers unprecedented insight into the regeneration of **3344-OAc**, but has additionally produced several unforeseen results. Our sampling revealed that the vanadate ligand is far less inert than previously thought.^52^ Having the ability to participate in the formation of surprisingly stable JT-distorted bonds, this hexadentate ligand even demonstrated an ability to additionally interact with the cubane core of the catalyst at the apical O1 atom, facilitated by three JT axes cooperatively pushing O1 toward the vanadate.

Equally unexpectedly, we found many strongly distorted or partially dissociated structures using our multistep sampling approach. Two types of degradation processes were identified: cubane opening and ligand dissociation. All barriers of investigated reactions were quite low, underlining the role of JT effects in facilitating the reactivity of the catalyst. Thus, in the **3333** and **3334** oxidation states, the catalyst in many ways appears to be less stable than at higher oxidation states; however, the majority of the investigated catalyst degradation reactions were found to be reversible. Only for specific ligand configurations in the **3333** oxidation state, some forms of ligand dissociation reactions may be irreversible. Most interestingly, a unique degradation product was discovered, which is most likely formed by protonation of O4 by solvent water. While the kinetics of this process remain unknown, we argue that a fairly high kinetic barrier is required to explain the high turnover observed in the experiments,^46^ as the O4-protonated structure is more stable than the deactivated catalyst **3333-o-o**. Nevertheless, this unusual structure offers a first glimpse at how a loss of catalytic activity over time could occur in **3344-OAc**.

Looking to the future, greater understanding of the fundamental principles involved in catalyst degradation could be achieved through simulations in explicit solvation, thereby contributing to the goal of imbuing this highly efficient molecular catalyst with greater stability. From an experimental point of view, the proposal of a complete catalytic cycle for **3344-OAc** opens up many avenues for further investigation, both to verify the accuracy of our proposal as well as to operationalize the insights gained from these simulations. Chief among these is the importance of JT effects for increasing the water oxidation activity of catalysts that include d^4^ metal centers. This structure–property relationship is already well-known in heterogeneous catalysis,^42−44,51^ and it is high time it were applied to the development of molecular catalysts for artificial water splitting.

## Supporting Information Available

Analysis of heuristics for sampling target selection, tables of stable isomers, interatomic distance analysis, and nudged elastic band simulation results (PDF)
Cartesian coordinates of relevant structures (PDF)
