# Supplementary material for: Regeneration and Degradation in a Biomimetic Polyoxometalate Water Oxidation Catalyst
Source: ACS Catal. 2023 Feb 14;13(5):3007–19. doi: 10.1021/acscatal.2c06301 (PMC9990072; doi:10.1021/acscatal.2c06301)
Supplement: Supplementary file 1 — cs2c06301_si_001.pdf [file cs2c06301_si_001.pdf]

Supporting Information:

Regeneration and Degradation in a Biomimetic Polyoxometalate Water Oxidation Catalyst

Ludwig Schwiedrzik,[a,b,*] Tina Rajkovic,[a] Leticia González[a,*]

[a] Institute of Theoretical Chemistry, Faculty of Chemistry, University of Vienna, Währinger Straße 17, 1090 Vienna, Austria

[b] Vienna Doctoral School in Chemistry (DoSChem), University of Vienna, Währinger Str. 42, 1090 Vienna, Austria

[*] ludwig.schwiedrzik@univie.ac.at, leticia.gonzalez@univie.ac.at

Contents

I.	Analysis of heuristics for sampling target selection	S2
II.	Tables of stable isomers	S4
III.	Interatomic distance analysis	S12
IV.	Nudged elastic band simulation results	S16

I. Analysis of heuristics for sampling target selection

In selecting which structures to target for sampling, we made use of a set of heuristic rules derived by Mai et al. for the relative stability of Jahn-Teller (JT) and redox isomers of the **3344-OAc** catalyst and its derivatives.[1] These rules were developed on the basis of optimized structures covering all oxidation states of the catalyst (**3333**, **3334**, **3344**, **3444**, **4444**) with one H_2O and one OH ligand. They take the form of absolute energy values for each type of JT axis that a structure might contain:

1. JT axes at MnA cost 7.5 kcal/mol.
2. A crossing of two JT axes costs 5 kcal/mol.
3. JT axes to the vanadate ligand cost 12 kcal/mol.
4. A crossing of three JT axes costs 17 kcal/mol.

In practice, these rules allow us to predict the energy of every conceivable isomer (relative to a structure without JT distortions) by adding up the respective value for every JT axis in that isomer. For example, **z444-OH-H_2O** has a predicted energy of 7.5 kcal/mol, while **z44y-OH-H_2O** has a predicted relative energy of 24.5 kcal/mol. We thus created an energy ranking of all isomers for each oxidation state of the catalyst. In the case of the **4444** and **3444** oxidation states, the total number of symmetry-unique isomers is small enough so that all of them could be sampled. For the **3344**, **3334**, and **3333** oxidation states, we decided to limit our sampling to those isomers within less than 12 kcal/mol of the most stable isomer for each oxidation state, thereby greatly reducing the computational cost of our study.

Having obtained a large number of optimized geometries and their respective Gibbs energies, we now wanted to evaluate the utility and accuracy of the heuristic-based target selection by comparing the predicted energies obtained using the heuristic rules E_{pred} to the calculated energies from our DFT calculations E_{calc}. To this end, we split the optimized geometries into six groups according to their ligand configurations: **o-o** contains all structures listed in table S2; **H_2O-o/o-H_2O** contains all structures from tables S3 and S4; **OH-o/o-OH** contains all structures from tables S5 and S6; **OH-H_2O** contains all structures listed in table S7; **PCET 1** contains all structures from table S8; and **PCET 2** contains all structures from table S9 (see section II). Within each of these groups, the most stable isomer belonging to a specific ligand configuration and oxidation state is used as a reference for that combination of ligand configuration and oxidation state by defining its E_{calc} as equal to its E_{pred}. Other structures of the same ligand configuration and oxidation state then have their E_{calc} defined relative to this reference, allowing us to compare values across multiple oxidation states and ligand configurations. These reference structures are omitted in the linear fits shown in figure S1, as they have an absolute error of 0.0 kcal/mol by definition.

Figure S1 shows linear fits obtained by plotting E_{pred} vs E_{calc} for each of the six groups of structures, while Table S1 shows the fit parameters: slope, intercept, R^2, and the mean absolute error (MAE). Ideally, these linear fits should have a slope of 1, an intercept of 0, $R^2 = 1.0$, and MAE = 0.0; this would indicate that the heuristics-derived energies perfectly predict the DFT-calculated energies. Deviations from these ideal values allow us to evaluate the accuracy and precision of the heuristic predictions.

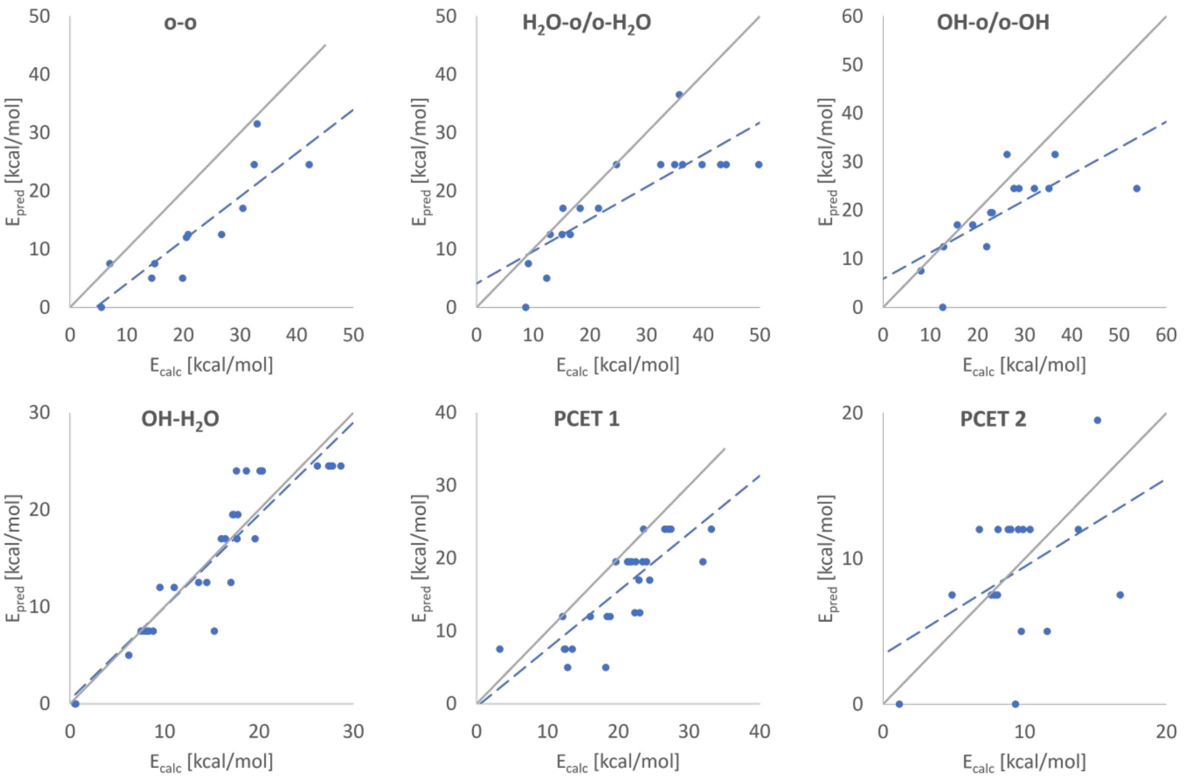

Figure S1: Linear fits of heuristics-predicted E_{pred} vs DFT-calculated E_{calc} (in kcal/mol) for all six groups of structures. The blue dashed lines represent the linear fits, while the grey unbroken line represents an ideal fit with a slope of 1 and intercept of 0.

Table S1: Parameters of the linear fits shown in figure S1: number of structures, slope, intercept, R^2, and MAE (in kcal/mol). In the latter four categories, the values closest to an ideal fit are highlighted in bold.

Group	**o-o**	**H_2O-o/ o-H_2O**	**OH-o/ o-OH**	**OH-H_2O**	**PCET 1**	**PCET 2**
Structures	12	18	15	31	29	18
Slope	0,75	0,55	0,54	**0,95**	0,79	0,60
Intercept	-3,37	4,06	5,88	0,45	**-0,43**	3,40
R^2	0,76	0,68	0,52	**0,86**	0,66	0,20
MAE	10,94	14,23	9,82	7,02	14,61	**6,24**

For all six groups of structures, the linear fits show that the predicted energies are able to at least qualitatively reproduce the calculated energies: all slopes are positive, showing that the energy ranking from low- to high-energy structure is comparable between predicted and calculated energy values. Non-zero intercept values are a lesser concern, as they represent an overall bias in the prediction affecting all structures equally and therefore not interfering with the correct energy ranking. In all cases, the energies of less stable structures with a greater number of JT axes are at least slightly underestimated, shown by the downward deviation of the linear fits (blue dashed lines) from the ideal fit (grey unbroken line) in figure S1. A small degree of overestimation of relative energies can be observed for structures under 10 kcal/mol calculated energy in the **H_2O-o/o-H_2O**, **OH-o/o-OH**, and **PCET 2** groups, but as only structures occupying the higher oxidation states of the catalyst (of which all possible isomers

were sampled) occupy this energy range, this overestimation does not reflect negatively on the practical accuracy of our heuristics. The highest precision is achieved in the **OH-H_2O** group, which was to be expected as this is the ligand configuration the heuristic rules were developed for. As already noted by Mai et al., the energy "costs" associated with each type of JT axis are heavily dependent on the ligand configuration of the system.[1] This observation is confirmed by the significantly lower R^2 values obtained for the other five groups of structures. Finally, the MAE values allow us to quantify the error separating predicted and calculated energies in terms of a mean absolute deviation in kcal/mol for each of the groups of structures. Again, the **OH-H_2O** group has the lowest value among the first four groups, which each cover all investigated oxidation states of the catalyst. The MAE of the **PCET 2** group is even lower, but this is mainly because this group only contains structures from the **3344**, **3444**, and **4444** oxidation states bearing fewer JT axes.

II. Tables of stable isomers

In the following, a table for each ligand configuration in the different oxidation states is provided, collecting relative Gibbs energies, relative Boltzmann populations at thermal equilibrium (T = 298.15 K) and spin populations.

Table S2: Unique isomers for the ligand configuration with two open coordination sites (**o-o**), grouped according to the overall oxidation state of the cubane core, charge q and multiplicity 2S+1. Columns from left to right: Oxidation state of the cubane core; name of isomer; Gibbs free energy relative to **zzzz-o-o*** (in eV); relative Boltzmann population at thermal equilibrium (T = 298.15 K); spin populations of MnA-D. Further notes: other spin populations over 0.15 with their atom name and number, † = structure obtained by applying two constraints along at least one JT axis. Structures with Boltzmann populations over 5.0% are highlighted in bold.

Ox. state	Isomer	ΔG_{rel} [eV]	Pop.	Spin populations MnA	MnB	MnC	MnD	Notes
4444	q = 0	2S+1 = 13						
	**4444-o-o**	21.19	**100%**	3.04	2.95	2.85	2.85	
3444	q = -1	2S+1 = 14						
	z444-o-o	14.79	0.0%	3.95	2.97	2.93	2.93	†
	444x-o-o	14.73	0.0%	3.03	2.97	2.92	3.86	†
	**4z44-o-o**	14.49	**100%**	3.04	3.88	2.92	2.92	†
3344	q = -2	2S+1 = 15						
	44yz-o-o	9.28	0.0%	3.01	2.97	3.86	3.86	†
	44yx-o-o	9.25	0.0%	3.02	2.93	3.86	3.86	†
	z44x-o-o	9.04	0.0%	3.89	2.98	2.98	3.89	†
	4z4x-o-o	9.02	0.0%	3.03	3.86	2.96	3.85	†
	**zz44-o-o**	8.72	**100%**	3.90	3.89	2.99	2.99	†
3334	q = -3	2S+1 = 16						
	4zyz-o-o*	4.43	0.0%	3.00	3.84	3.86	3.87	O1 0.17, †
	z4yx-o-o	4.27	0.0%	3.89	2.94	3.89	3.89	†
	**zz4x-o-o**	4.01	**9.7%**	3.90	3.87	3.00	3.88	O1 0.15, †
	**zz4z-o-o***	3.95	**90.3%**	3.93	3.88	3.03	3.87	†
3333	q = -4	2S+1 = 17						

	zzyx-o-o*	0.46	0.0%	3.96	3.93	3.87	3.87	O1 0.25
	**zzzz-o-o**	0.06	**5.5%**	3.91	3.87	3.88	3.88	†
	**zzyz-o-o***	0.04	**26.3%**	3.91	3.86	3.90	3.87	O1 0.18, †
	**zzzz-o-o***	0.00	**68.2%**	3.91	3.92	3.88	3.88	†

Table S3: Unique isomers for the ligand configuration with H_2O bound to MnB and and open coordination site at MnA (**H_2O-o**), grouped according to the overall oxidation state of the cubane core, charge q and multiplicity 2S+1. Columns from left to right: Oxidation state of the cubane core; name of isomer; Gibbs free energy relative to **zzzz-o-o*** (in eV); relative Boltzmann population at thermal equilibrium (T = 298.15 K); spin populations of MnA-D. Further notes: other spin populations over 0.15 with their atom name and number, † = structure obtained by applying two constraints along at least one JT axis. Structures with Boltzmann populations over 5.0% are highlighted in bold.

Ox. state	Structure	ΔG_{rel} [eV]	Pop.	Spin populations MnA	MnB	MnC	MnD	Notes
4444	q = 0	2S+1 = 13						
	**4444-H_2O-o**	20.22	**100%**	3.04	2.93	2.87	2.87	
3444	q = -1	2S+1 = 14						
	**444x-H_2O-o**	14.00	**21.4%**	3.04	2.98	2.94	3.86	
	**z444-H_2O-o**	13.95	**78.6%**	3.90	2.96	2.95	2.95	
3344	q = -2	2S+1 = 15						
	**z4y4-H_2O-o**	8.51	**100%**	3.90	3.00	3.89	2.99	
3334	q = -3	2S+1 = 16						
	**zz4x-H_2O-o**	3.96	**46.9%**	3.90	3.89	3.00	3.89	
	**z4yx-H_2O-o**	3.94	**53.1%**	3.90	2.98	3.89	3.89	
3333	q = -4	2S+1 = 17						
	zzxz-H_2O-o*	0.54	0.0%	3.92	3.87	3.87	3.88	O2 0.20
	zxyx-H_2O-o	0.50	0.0%	3.94	3.86	3.89	3.90	O1 0.15
	zzyx-H_2O-o*	0.39	0.0%	3.95	3.84	3.87	3.87	O1 0.31
	**zzzx-H_2O-o***	0.05	**42.6%**	3.91	3.86	3.87	3.90	O1 0.19
	**xzxx-H_2O-o***	0.05	**57.4%**	3.93	3.87	3.87	3.85	O1 0.22

Table S4: Unique isomers for the ligand configuration with an open coordination site at MnB and H_2O bound to MnA (**o-H_2O**), grouped according to the overall oxidation state of the cubane core, charge q and multiplicity 2S+1. Columns from left to right: Oxidation state of the cubane core; name of isomer; Gibbs free energy relative to **zzzz-o-o*** (in eV); relative Boltzmann population at thermal equilibrium (T = 298.15 K); spin populations of MnA-D. Further notes: other spin populations over 0.15 with their atom name and number, † = structure obtained by applying two constraints along at least one JT axis. Structures with Boltzmann populations over 5.0% are highlighted in bold.

Ox. state	Structure	ΔG_{rel} [eV]	Pop.	Spin populations MnA	MnB	MnC	MnD	Notes

4444	q = 0	2S+1 = 13						
	**4444-o-H_2O**	20.11	**100%**	3.04	2.95	2.88	2.88	
3444	q = -1	2S+1 = 14						
	**4z44-o-H_2O**	13.57	**100%**	3.04	3.88	2.94	2.94	
3344	q = -2	2S+1 = 15						
	44yx-o-H_2O	8.70	0.0%	3.04	2.93	3.87	3.87	
	xz44-o-H_2O	8.56	0.1%	3.91	3.91	2.99	3.00	
	**4z4x-o-H_2O**	8.38	**99.9%**	3.04	3.86	2.97	3.86	O1 0.15
3334	q = -3	2S+1 = 16						
	4zyx-o-H_2O*	4.32	0.0%	3.04	3.80	3.84	3.84	O1 0.34
	4zxx-o-H_2O	4.17	0.0%	3.00	3.85	3.88	3.87	O1 0.16
	xz4x-o-H_2O	4.10	0.1%	3.92	3.88	3.01	3.89	O1 0.15
	4zzx-o-H_2O	4.04	1.0%	3.01	3.84	3.88	3.86	O1 0.17
	xzy4-o-H_2O	4.04	1.2%	3.91	3.88	3.89	3.03	
	**zzy4-o-H_2O**	3.92	**97.7%**	3.92	3.87	3.89	3.01	O1 0.16
3333	q = -4	2S+1 = 17						
	xzyx-o-H_2O*	0.72	0.0%	3.92	3.93	3.88	3.87	O1 0.24
	xzyy-o-H_2O	0.47	0.0%	3.92	3.94	3.90	3.89	
	xzxx-o-H_2O	0.43	0.0%	3.92	3.87	3.87	3.89	O1 0.19
	xzyz-o-H_2O*	0.29	0.0%	3.95	3.92	3.90	3.89	
	zzzx-o-H_2O*	0.14	0.0%	3.93	3.91	3.87	3.90	O1 0.17
	**xzzx-o-H_2O***	-0.37	**100%**	3.91	3.90	3.87	3.89	O1 0.15

Table S5: Unique isomers for the ligand configuration with an open coordination site at MnB and OH bound to MnA (**o-OH**), grouped according to the overall oxidation state of the cubane core, charge q and multiplicity 2S+1. Columns from left to right: Oxidation state of the cubane core; name of isomer; Gibbs free energy relative to **zzzz-o-o*** (in eV); relative Boltzmann population at thermal equilibrium (T = 298.15 K); spin populations of MnA-D. Further notes: other spin populations over 0.15 with their atom name and number, † = structure obtained by applying two constraints along at least one JT axis. Structures with Boltzmann populations over 5.0% are highlighted in bold.

Ox. state	Structure	ΔG_{rel} [eV]	Pop.	Spin populations				Notes
				MnA	MnB	MnC	MnD	
4444	q = -1	2S+1 = 13						
	**4444-o-OH**	20.23	**100%**	2.94	2.97	2.92	2.91	
3444	q = -2	2S+1 = 14						
	**4z44-o-OH**	14.04	**100%**	2.97	3.89	2.98	2.98	
3344	q = -3	2S+1 = 15						
	yz44-o-OH	9.41	0.6%	3.88	3.91	3.02	3.02	
	**4zy4-o-OH**	9.28	**99.4%**	3.02	3.87	3.88	2.99	O1 0.16
3334	q = -4	2S+1 = 16						

	4zxx-o-OH	5.53	0.0%	3.02	3.86	3.87	3.88	O1 0.16
	4zyz-o-OH	5.39	0.3%	3.01	3.85	3.88	3.89	O1 0.17
	**yzy4-o-OH**	5.26	**36.9%**	3.94	3.89	3.90	3.03	O1 0.16
	**yz4x-o-OH**	5.25	**62.9%**	3.93	3.89	3.04	3.90	
3333	q = -5	2S+1 = 17						
	yzyx-o-OH*	2.05	0.1%	3.95	3.85	3.89	3.89	O1 0.28
	xzxx-o-OH	2.00	0.4%	3.93	3.88	3.88	3.89	O1 0.18
	xzxz-o-OH	1.94	4.9%	3.91	3.92	3.89	3.90	
	**xzyz-o-OH**	1.86	**94.6%**	3.93	3.86	3.90	3.90	O1 0.16

Table S6: Unique isomers for the ligand configuration with OH bound to MnB and an open coordination site at MnA (**OH-o**), grouped according to the overall oxidation state of the cubane core, charge q and multiplicity 2S+1. Columns from left to right: Oxidation state of the cubane core; name of isomer; Gibbs free energy relative to **zzzz-o-o*** (in eV); relative Boltzmann population at thermal equilibrium (T = 298.15 K); spin populations of MnA-D. Further notes: other spin populations over 0.15 with their atom name and number, † = structure obtained by applying two constraints along at least one JT axis. Structures with Boltzmann populations over 5.0% are highlighted in bold.

Ox. state	Structure	ΔG_{rel} [eV]	Pop.	Spin populations				Notes
				MnA	MnB	MnC	MnD	
4444	q = -1	2S+1 = 13						
	**4444-OH-o**	20.35	**100%**	3.04	2.90	2.91	2.91	
3444	q = -2	2S+1 = 14						
	444x-OH-o	14.67	0.0%	3.04	2.95	2.96	3.86	
	**z444-OH-o**	14.45	**100%**	3.90	2.94	2.98	2.98	
3344	q = -3	2S+1 = 15						
	**z44x-OH-o**	9.50	**100%**	3.90	2.99	3.01	3.89	
3334	q = -4	2S+1 = 16						
	zxy4-OH-o	5.60	0.2%	3.89	3.89	3.90	3.03	
	zx4x-OH-o	5.59	0.4%	3.94	3.88	3.03	3.90	
	z4yx-OH-o	5.55	0.8%	3.91	3.03	3.87	3.87	O1 0.15
	**z4yz-OH-o**	5.44	**98.6%**	3.93	3.05	3.91	3.88	
3333	q = -5	2S+1 = 17						
	zzyx-OH-o*	2.84	0.0%	3.95	3.87	3.88	3.88	O1 0.19
	zxzx-OH-o*	2.09	0.0%	3.89	3.91	3.88	3.89	
	zxyx-OH-o*	2.03	0.1%	3.92	3.91	3.88	3.85	O1 0.18
	**yyyx-OH-o***	1.90	**28.9%**	3.89	3.91	3.84	3.88	O1 0.18
	**xxxx-OH-o***	1.88	**71.0%**	3.90	3.89	3.89	3.87	

Table S7: Unique isomers for the ligand configuration with an OH and an H_2O ligand, grouped according to the overall oxidation state of the cubane core, charge q and multiplicity 2S+1. Columns from left to right: Oxidation state of the cubane core; name of isomer; Gibbs free energy relative to **zzzz-o-o*** (in eV); relative Boltzmann population at thermal equilibrium (T = 298.15 K); spin populations of MnA-D. Further notes: other spin populations over 0.15 with their atom name and number, † = structure obtained by applying two constraints along at least one JT axis. Structures with Boltzmann populations over 5.0% are highlighted in bold.

Ox. state	Structure	ΔG_{rel} [eV]	Pop.	Spin populations MnA	MnB	MnC	MnD	Notes
	q = -1	2S+1 = 13						
4444	**4444-H_2O-OH**	19.28	**29.6%**	2.98	2.94	2.93	2.93	
	**4444-OH-H_2O**	19.26	**70.4%**	3.03	2.93	2.93	2.93	
	q = -2	2S+1 = 14						
	x444-OH-H_2O	14.04	0.0%	3.91	2.98	2.98	2.98	
3444	z444-OH-H_2O	14.03	0.0%	3.91	2.97	2.99	2.99	
	**444x-H_2O-OH**	13.71	**41.9%**	3.03	2.99	2.99	3.88	
	**4z44-H_2O-OH**	13.69	**58.1%**	3.01	3.88	2.99	2.99	†
	q = -3	2S+1 = 15						
	zz44-OH-H_2O	9.50	0.0%	3.92	3.86	3.03	3.03	
	44xx-OH-H_2O	9.31	0.0%	3.02	3.01	3.86	3.88	†
	4x4x-OH-H_2O	9.25	0.0%	3.03	3.88	2.99	3.88	†
	z44x-OH-H_2O	9.21	0.1%	3.92	3.02	3.02	3.90	
3344	x44x-H_2O-OH	9.19	0.3%	3.90	3.03	3.03	3.90	
	y44x-H_2O-OH	9.17	0.8%	3.91	3.03	3.03	3.90	
	xz44-H_2O-OH	9.16	1.3%	3.89	3.92	3.02	3.02	
	**44yx-H_2O-OH**	9.10	**5.9%**	3.05	2.98	3.88	3.88	
	**4z4x-H_2O-OH**	9.05	**91.5%**	3.04	3.88	2.99	3.88	
	q = -4	2S+1 = 16						
	4xxx-H_2O-OH*	5.47	0.0%	3.02	3.87	3.88	3.89	†
	4xyy-H_2O-OH	5.46	0.0%	3.02	3.88	3.88	3.89	†
	4zyx-H_2O-OH*	5.44	0.0%	3.07	3.85	3.85	3.85	O1 0.28
	4zzz-H_2O-OH	5.40	0.0%	3.01	3.88	3.88	3.88	†
	zx4x-OH-H_2O	5.36	0.0%	3.92	3.89	3.03	3.90	†
	4zxx-H_2O-OH	5.36	0.0%	3.03	3.88	3.88	3.87	†
	4xzx-H_2O-OH	5.35	0.0%	3.03	3.88	3.89	3.88	†
3334	zy4x-OH-H_2O	5.34	0.0%	3.92	3.90	3.04	3.91	†
	x4yz-OH-H_2O	5.34	0.0%	3.92	3.06	3.90	3.90	†
	z4yx-OH-H_2O	5.33	0.0%	3.93	3.04	3.89	3.89	†
	4xyx-H_2O-OH	5.30	0.1%	3.04	3.87	3.88	3.87	†
	4zyz-H_2O-OH	5.28	0.2%	3.03	3.87	3.87	3.88	†
	x4yx-H_2O-OH	5.22	3.0%	3.92	3.01	3.90	3.90	
	**xz4x-H_2O-OH**	5.18	**12.7%**	3.91	3.91	3.02	3.90	†
	**yz4x-H_2O-OH**	5.13	**83.8%**	3.91	3.91	3.03	3.90	

	q = -5	2S+1 = 17						
	zxyx-OH-H_2O*	2.09	0.1%	3.92	3.89	3.89	3.88	†
	yzyx-H_2O-OH*	2.05	0.4%	3.93	3.87	3.88	3.88	O1 0.25
3333	yzyy-H_2O-OH*	2.04	0.5%	3.91	3.88	3.89	3.87	O1 0.18, †
	xzyy-H_2O-OH*	2.03	0.7%	3.91	3.89	3.89	3.88	O1 0.16, †
	**xxyx-H_2O-OH**	1.98	**5.5%**	3.93	3.87	3.90	3.89	O1 0.16, †
	**yzzx-H_2O-OH***	1.91	**92.8%**	3.93	3.86	3.90	3.90	O1 0.16, †

Table S8: Unique isomers from the first PCET pathway, comprising those with two OH ligands, those with one oxo and one OH ligand, and those with two oxo ligands; grouped according to the overall oxidation state of the cubane core, charge q and multiplicity 2S+1. Columns from left to right: Oxidation state of the cubane core; name of isomer; Gibbs free energy relative to **zzzz-o-o*** (in eV); relative Boltzmann population at thermal equilibrium (T = 298.15 K); spin populations of MnA-D. Further notes: other spin populations over 0.15 with their atom name and number, † = structure obtained by applying two constraints along at least one JT axis. Structures with Boltzmann populations over 5.0% are highlighted in bold.

Ox. state	Structure	ΔG_{rel} [eV]	Pop.	Spin populations				Notes
				MnA	MnB	MnC	MnD	
	q = -5	2S+1 = 14						
3444	x444-O-O	19.80	0.4%	3.76	2.71	3.04	3.04	O5 0.35, O6 0.17
	**444x-O-O**	19.66	**99.6%**	2.71	2.67	3.01	3.89	O5 0.39, O6 0.38, †
	q = -5	2S+1 = 15						
	xx44-OH-O	13.70	0.0%	3.77	3.87	3.02	2.99	O6 0.15, †
	44xx-O-OH	13.59	0.0%	3.02	2.75	3.87	3.87	O5 0.30, †
	4x4x-O-OH	13.58	0.0%	3.01	3.78	3.00	3.89	†
	44yx-O-OH	13.57	0.0%	3.03	2.76	3.85	3.85	O1 0.18, O5 0.30
3344	44yz-O-OH	13.47	0.0%	3.02	2.76	3.88	3.87	O5 0.30, †
	x44x-OH-O	13.36	2.7%	3.78	3.03	3.04	3.91	
	44yx-OH-O	13.33	4.1%	2.76	3.04	3.88	3.88	O6 0.33
	**y44x-O-OH**	13.32	**14.4%**	3.90	2.77	3.02	3.90	O5 0.29, †
	**x44x-O-OH**	13.31	**17.5%**	3.90	2.77	3.02	3.90	O5 0.29
	**4x4x-OH-O**	13.30	**26.7%**	2.76	3.88	3.03	3.90	O6 0.30, †
	**4y4x-OH-O**	13.30	**34.4%**	2.76	3.90	3.03	3.90	O6 0.31, †

	q = -5	2S+1 = 16						
3334	4zzz-HO-HO	7.68	0.0%	3.01	3.87	3.87	3.87	†
	xz4z-HO-HO	7.63	0.0%	3.90	3.88	3.03	3.88	†
	4xyy-OH-OH	7.44	0.0%	3.01	3.90	3.88	3.89	†
	4xyy-HO-HO	7.42	0.0%	3.04	3.91	3.88	3.89	†
	4xxx-OH-OH*	7.40	0.0%	3.02	3.89	3.88	3.88	†
	4xxx-HO-HO*	7.40	0.0%	3.03	3.89	3.88	3.89	†
	4xyx-OH-OH	7.31	0.0%	3.04	3.89	3.87	3.86	†
	x4xx-OH-OH	7.29	0.0%	3.91	3.06	3.88	3.89	†
	4xzx-HO-HO	7.27	0.1%	3.03	3.91	3.88	3.89	†
	x4xx-HO-HO	7.26	0.1%	3.91	3.06	3.88	3.90	†
	y4yx-OH-OH	7.25	0.1%	3.92	3.05	3.88	3.89	O1 0.16
	4yyx-HO-HO	7.24	0.2%	3.04	3.89	3.87	3.88	†
	yy4x-OH-OH	7.22	0.4%	3.90	3.90	3.04	3.91	†
	y4yx-HO-HO	7.22	0.4%	3.91	3.05	3.89	3.89	O1 0.16
	x4yz-OH-OH	7.20	0.9%	3.92	3.05	3.90	3.90	†
	xx4x-HO-HO	7.19	1.4%	3.90	3.88	3.03	3.90	†
	x4yz-HO-HO	7.17	2.5%	3.93	3.05	3.90	3.90	†
	**xx4x-OH-OH**	7.10	**40.1%**	3.91	3.88	3.03	3.90	†
	**yy4x-HO-HO**	7.09	**53.7%**	3.92	3.89	3.04	3.92	†

Table S9: Unique isomers from the second PCET pathway, comprising those with two OH ligands, those with one oxo and one OH ligand, and those with two oxo ligands; grouped according to the overall oxidation state of the cubane core, charge q and multiplicity 2S+1. Columns from left to right: Oxidation state of the cubane core; name of isomer; Gibbs free energy relative to **zzzz-o-o*** (in eV); relative Boltzmann population at thermal equilibrium (T = 298.15 K); spin populations of MnA-D. Further notes: other spin populations over 0.15 with their atom name and number, † = structure obtained by applying two constraints along at least one JT axis. Structures with Boltzmann populations over 5.0% are highlighted in bold.

Ox. state	Structure	ΔG_{rel} [eV]	Pop.	Spin populations MnA	MnB	MnC	MnD	Notes
	q = -4	2S+1 = 13						
4444	**4444-O-O**	23.58	**100%**	2.63	2.61	3.02	3.02	O5 0.42<br>O6 0.43
	q = -4	2S+1 = 14						
3444	4z44-O-OH	17.31	0.0%	2.98	3.77	3.01	3.01	†
	4x44-OH-O	17.20	0.0%	2.69	3.87	3.01	3.02	O6 0.36, †
	y444-O-OH	17.12	0.0%	3.88	2.71	3.01	3.01	O5 0.32
	**444x-O-OH**	16.95	**12.6%**	3.00	2.70	2.99	3.88	O5 0.34
	**444x-OH-O**	16.90	**87.4%**	2.70	2.99	3.02	3.89	O6 0.37

	q = -4	2S+1 = 15						
3344	yz44-HO-HO	11.04	0.0%	3.88	3.87	3.04	3.04	†
	xx44-OH-OH	10.97	0.0%	3.89	3.87	3.01	3.02	†
	44xx-OH-OH	10.91	0.0%	3.02	3.00	3.87	3.88	†
	44yx-OH-OH	10.82	0.0%	3.04	3.01	3.86	3.86	
	44yz-OH-OH	10.76	0.2%	3.02	3.02	3.88	3.88	†
	4y4x-OH-OH	10.74	0.5%	3.02	3.88	3.02	3.89	†
	44yx-HO-HO	10.74	0.3%	3.02	3.02	3.87	3.87	
	44yz-HO-HO	10.73	1.0%	3.02	3.03	3.88	3.88	†
	4x4x-OH-OH	10.71	2.3%	3.02	3.87	3.01	3.89	†
	4y4x-HO-HO	10.70	3.2%	3.02	3.89	3.02	3.89	†
	**4x4x-HO-HO**	10.67	**10.8%**	3.01	3.88	3.02	3.89	†
	**x44x-OH-OH**	10.66	**11.3%**	3.90	3.03	3.03	3.90	
	**y44x-OH-OH**	10.66	**16.2%**	3.91	3.03	3.03	3.90	
	**x44x-HO-HO**	10.65	**23.9%**	3.90	3.03	3.03	3.90	
	**y44x-HO-HO**	10.64	**30.2%**	3.89	3.03	3.03	3.90	†

III. Interatomic distance analysis

In the following, histograms of the interatomic distances between each Mn center and every one of its coordinating atoms is provided. For the most part, these show a common pattern: two peaks representing bonds not lengthened by JT distortions and JT-distorted bonds, respectively, and outliers above 2.5 Å representing partly dissociated structures.

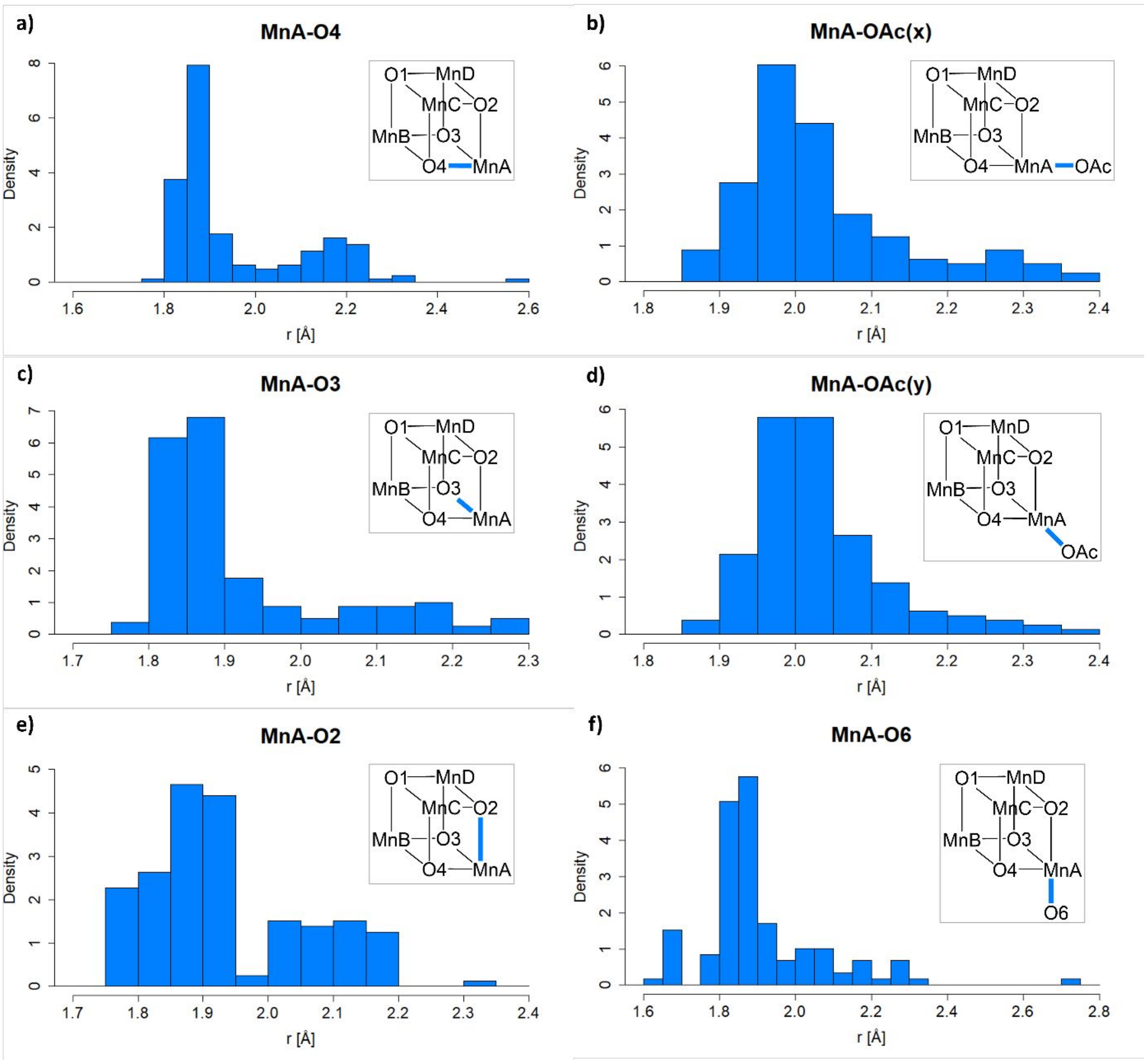

Figure S2: Interatomic distance distributions (in Å) for MnA and its coordination partners (bonds highlighted in blue in the insets). a) r(MnA-O4), b) r(MnA-OAc) along x axis, c) r(MnA-O3), d) r(MnA-OAc) along y axis, e) r(MnA-O2), f) r(MnA-O6).

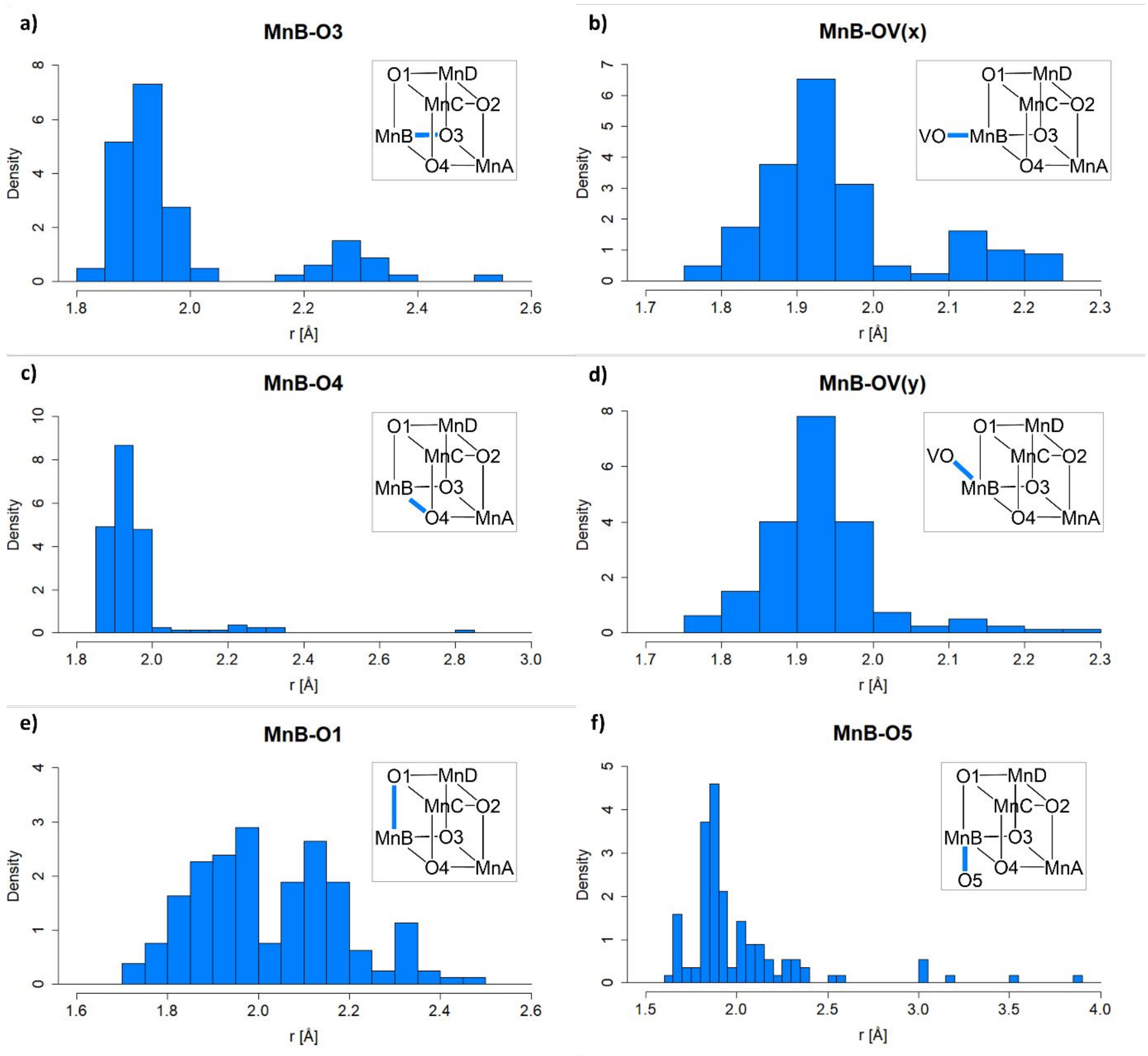

Figure S3: Interatomic distance distributions (in Å) for MnB and its coordination partners (bonds highlighted in blue in the insets). a) r(MnB-O3), b) r(MnB-OV) along x axis, c) r(MnB-O4), d) r(MnB-OV) along y axis, e) r(MnB-O1), f) r(MnB-O5).

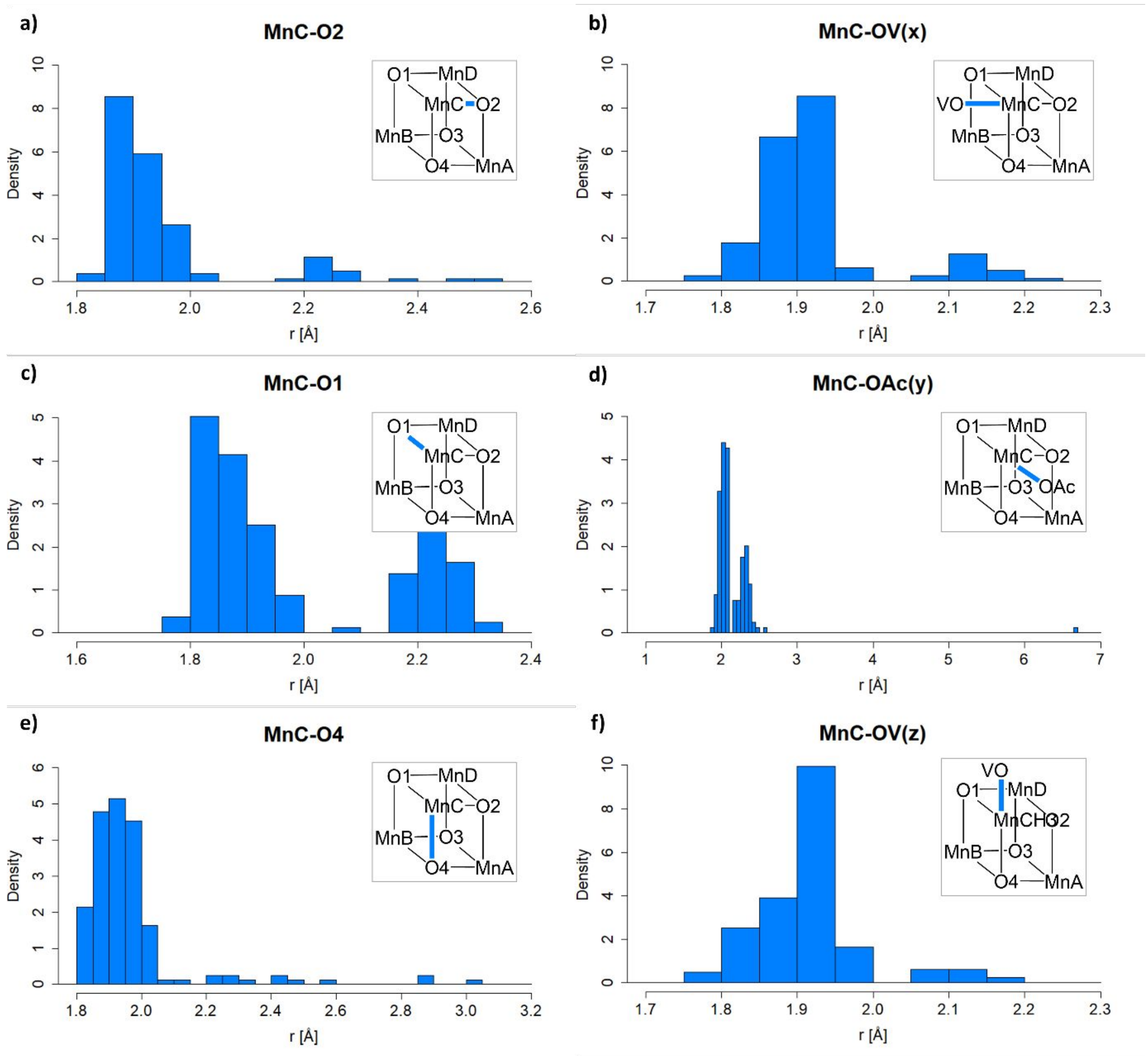

Figure S4: Interatomic distance distributions (in Å) for MnC and its coordination partners (bonds highlighted in blue in the insets). a) r(MnC-O2), b) r(MnC-OV) along x axis, c) r(MnC-O1), d) r(MnC-OAc) along y axis, e) r(MnC-O4), f) r(MnC-OV) along z axis.

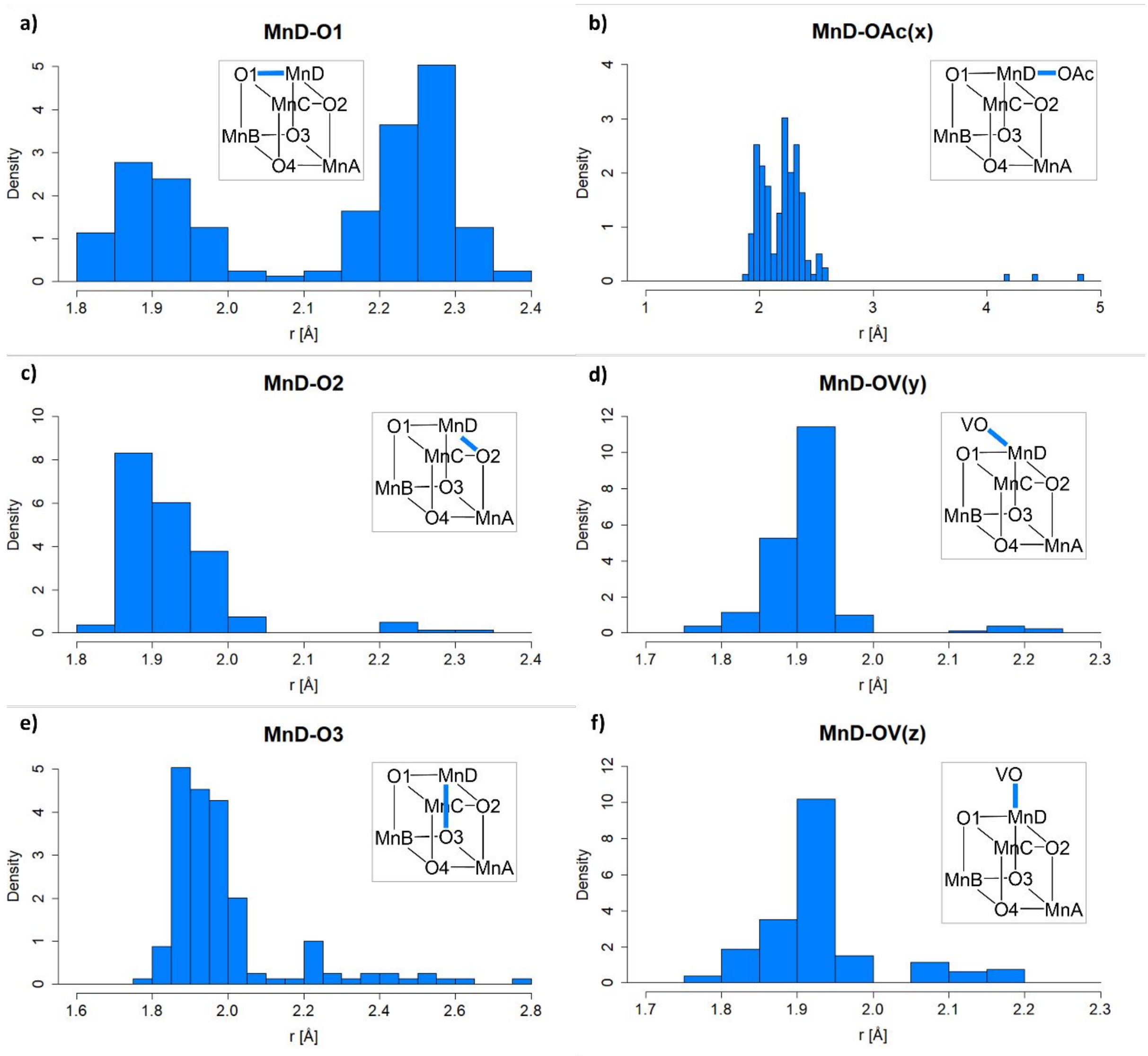

Figure S5: Interatomic distance distributions (in Å) for MnD and its coordination partners (bonds highlighted in blue in the insets). a) r(MnD-O1), b) r(MnD-OAc) along x axis, c) r(MnD-O2), d) r(MnD-OV) along y axis, e) r(MnD-O3), f) r(MnD-OV) along z axis.

IV. Nudged elastic band simulation results

In the following, figures summarizing the results of the nudged elastic band calculations NEB 1-6 are provided, showing the reactant and product structures, electronic energy changes along the minimum energy pathways, and significant bond distance changes along the minimum energy pathways indicating a reaction has taken place. Small changes in Mn-O interatomic distances (below 0.4 Å) typically represent a JT or redox isomerization, while larger changes point toward bond dissociations.

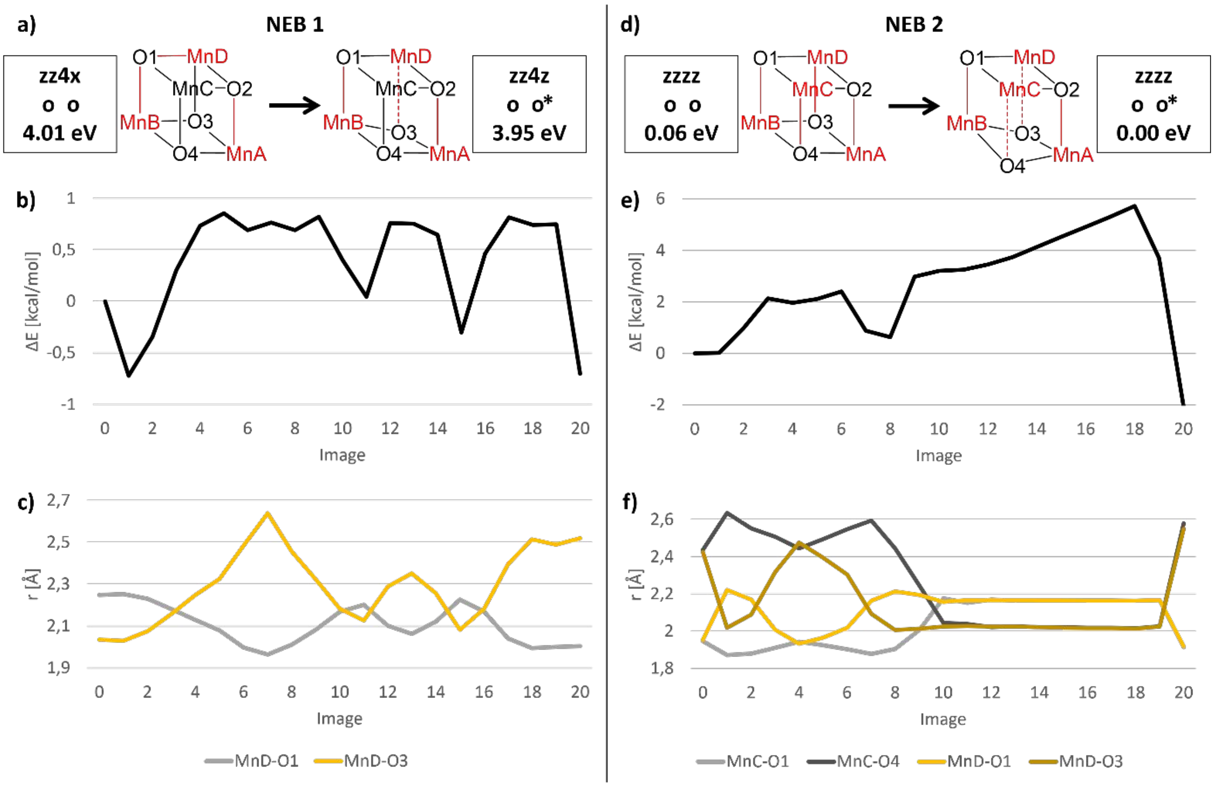

Figure S6: Results summary of the NEB 1 and NEB 2 calculations. a,d) Reactant and product structures with energies calculated at the B3LYP-D3/ZORA-def2-TZVP//B3LYP-D3/ZORA-def2-SVP level of theory. b,e) Energy changes along the minimum energy paths, calculated at the B3LYP-D3/ZORA-def2-SVP level of theory. c,f) Bond length changes along the minimum energy paths, in both cases showing fluctuation between two JT minima.

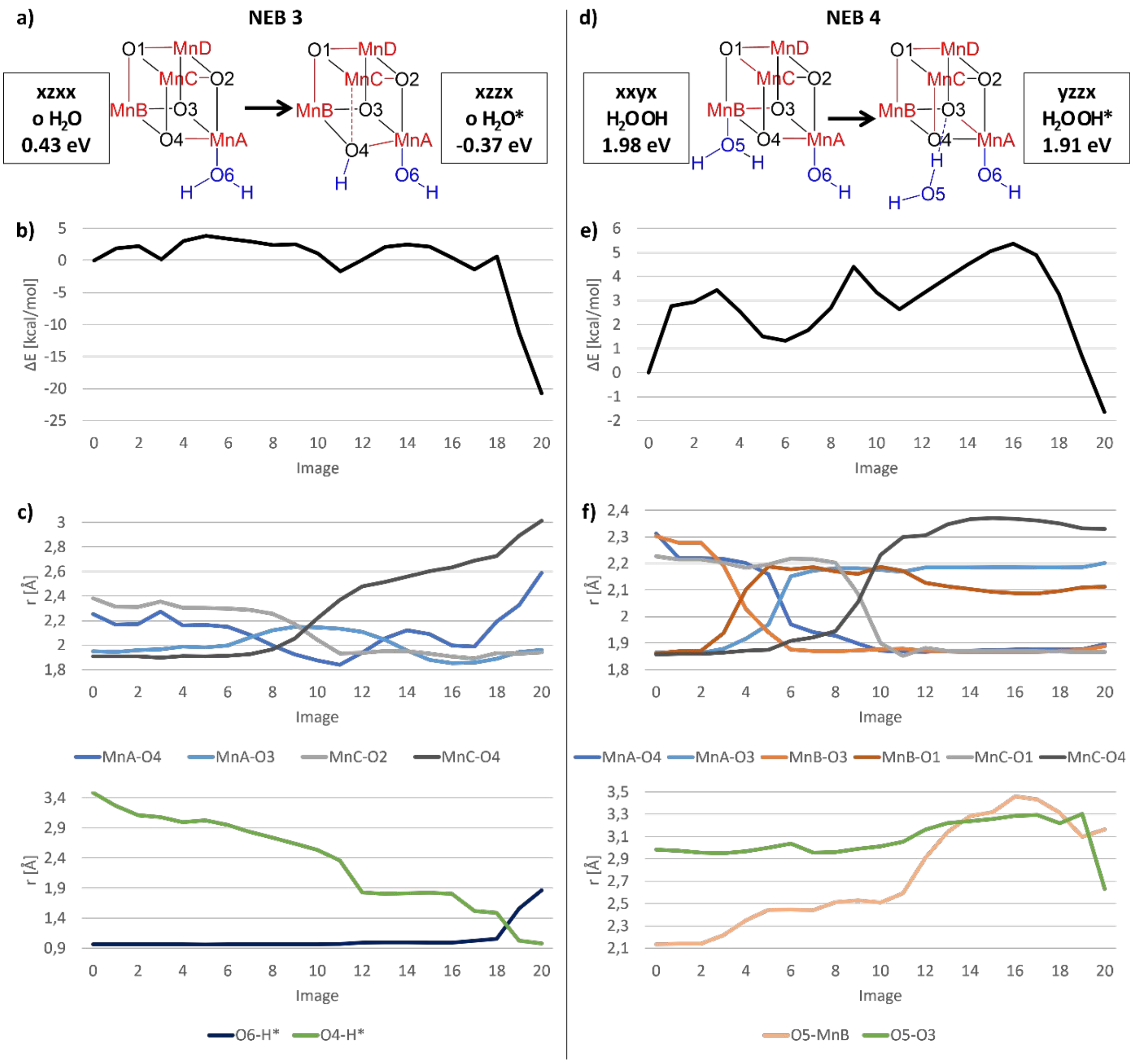

Figure S7: Results summary of the NEB 3 and NEB 4 calculations. a,d) Reactant and product structures with energies calculated at the B3LYP-D3/ZORA-def2-TZVP//B3LYP-D3/ZORA-def2-SVP level of theory. b,e) Energy changes along the minimum energy paths, calculated at the B3LYP-D3/ZORA-def2-SVP level of theory. c,f) Bond length changes along the minimum energy paths, in both cases the emergence of JT distortions in line with the studied degradation process can be observed before bond cleavage.

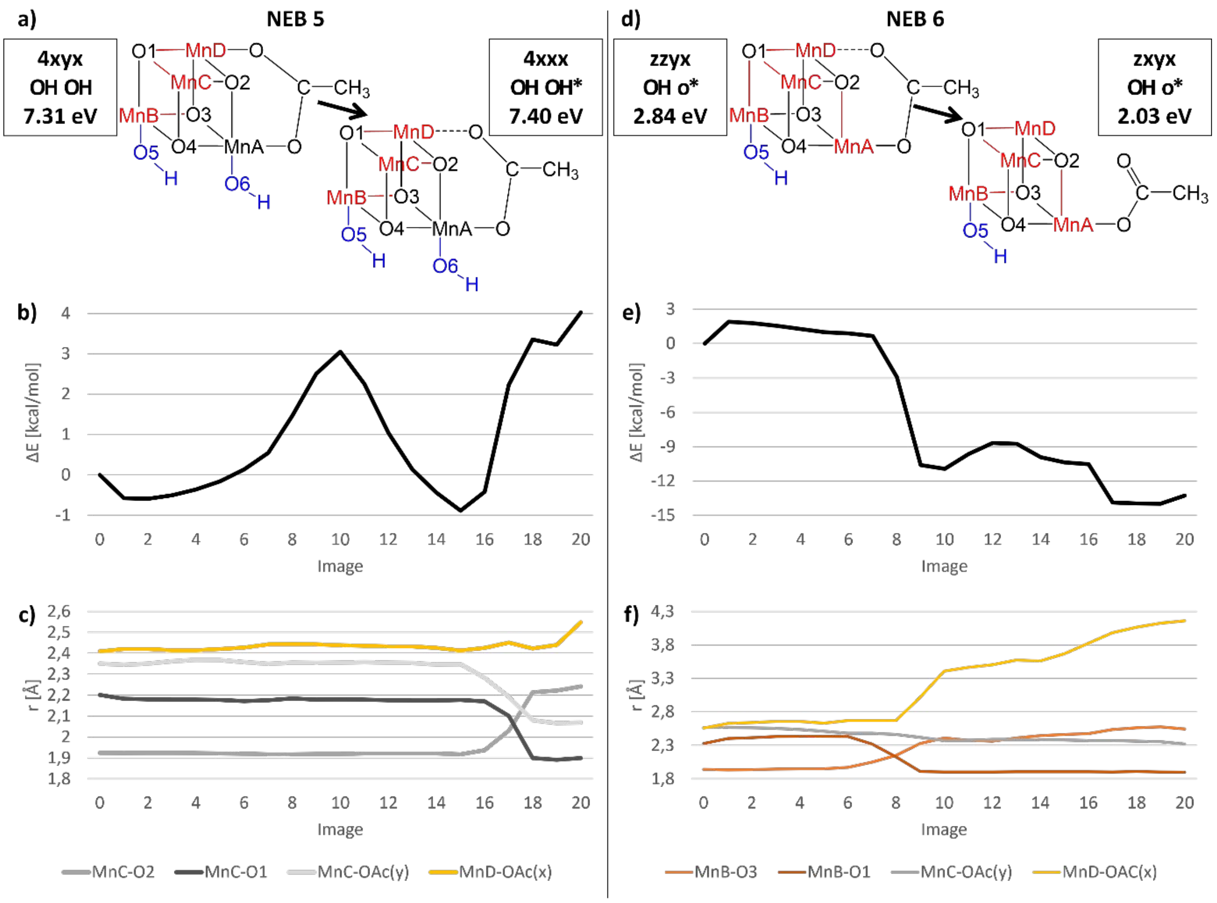

Figure S8: Results summary of the NEB 5 and NEB 6 calculations. a,d) Reactant and product structures with energies calculated at the B3LYP-D3/ZORA-def2-TZVP//B3LYP-D3/ZORA-def2-SVP level of theory. b,e) Energy changes along the minimum energy paths, calculated at the B3LYP-D3/ZORA-def2-SVP level of theory. c,f) Bond length changes along the minimum energy paths, in both cases JT distortions in line with the studied degradation are present in both the reactant and product structure.

Sources

(1) Mai, S.; Holzer, M.; Andreeva, A.; González, L. Jahn-Teller Effects in a Vanadate-Stabilized Manganese-Oxo Cubane Water Oxidation Catalyst. *Chemistry – A European Journal* **2021**, *27* (68), 17066–17077. https://doi.org/10.1002/chem.202102539.
