# Supplementary material for: Regeneration and Degradation in a Biomimetic Polyoxometalate Water Oxidation Catalyst
Source: ACS Catal. 2023 Feb 14;13(5):3007–19. doi: 10.1021/acscatal.2c06301 (PMC9990072; doi:10.1021/acscatal.2c06301)
Supplement: Supplementary file 2 — cs2c06301_si_002.pdf [file cs2c06301_si_002.pdf]

Supporting Information:

Regeneration and Degradation in a Biomimetic Polyoxometalate Water Oxidation Catalyst

Cartesian coordinates of selected intermediates

39

zz4x-o-o

Mn	-0.46043863259775	1.39757321890962	0.60346656355160
Mn	-2.54582391850981	-0.07598172968012	-0.63434577742565
Mn	0.05244959443181	-0.03556359441164	-1.86102347962476
Mn	-0.46376223984987	-1.55262368478516	0.67631785112536
O	-1.52929828801462	-0.05073389828715	1.13501595516685
O	-1.27062815960267	1.19011004162045	-1.18392345851841
O	0.80519614895211	0.20883753524482	0.09270312798035
O	-1.23082663432278	-1.29956058171656	-1.19135483073747
H	-4.33859737063273	-4.10883337087516	1.75061491614145
H	-5.39772079797277	-2.66673463422612	1.63578474987988
C	-3.50882995927472	-2.45269468407190	0.64433599139186
C	-4.67997986004749	-3.30008318146385	1.09931261747014
O	-2.35659311208091	-2.74952718801021	1.01479522183332
O	-3.81556253150914	-1.45082617844629	-0.10724567276389
H	-5.19830945460059	-3.71815197544459	0.22718080114644
O	-2.07144660147800	2.58051836950221	1.03416824401478
O	-3.70331374540236	1.38114674759181	0.03173669312333
O	1.13430462075361	1.39895797757899	-2.46202234948363
V	1.81828645291980	2.35476095754488	-1.19154313483982
O	2.40011466963571	3.68268330945607	-1.86735426051513
O	0.60772521857486	2.83203459413758	-0.03993471352877
O	0.79486928867926	-2.83335950229848	0.03202256957506
V	1.97755884636383	-2.37101309349291	-1.13602175590461
O	1.26580391719256	-1.39622294914084	-2.36967937737091
O	2.63484118039160	-3.67239097550439	-1.80582659651136
O	1.33254494183530	0.10752670065037	4.35463717906533

V 1.16274796800635 0.05827812897491 2.76514205105412

O 0.24924706084683 1.48073093481058 2.31730290274926

O 0.38652984773364 -1.43215472736830 2.38403158718462

O 2.85194253886434 0.13601844038477 2.08691495795428

O 5.16906156570386 0.11969130866402 0.77753153044279

V 3.59883214110930 0.09664382832853 0.50263320720016

O 3.30645423069676 -1.42488085570312 -0.33747801481719

O 3.28050831205786 1.53762609010051 -0.44779974779034

H -3.87904446987077 4.09029928350699 1.87559096572696

H -4.95517666388559 3.66734399864408 0.50149057278754

C -4.33347678747652 3.27088176322057 1.31323911189259

C -3.27771623052073 2.34777278968946 0.74719869145744

H -4.98867708709943 2.68742878636553 1.97337510991631

39

zz4z-o-o*

Mn -0.50592716417105 1.40479246892882 0.61572889504180

Mn -2.67659985148745 0.02250745918290 -0.75352728603884

Mn -0.06373089215375 -0.01668703516810 -1.92033871334603

Mn -0.54093463648382 -1.44973419001739 0.80385903969942

O -1.62560998413075 0.01157882351874 1.16456288311857

O -1.32159478414424 1.27059150055621 -1.18195012845483

O 0.61999038770837 0.03515402742378 0.12296790773745

O -1.49760149393176 -1.15553667110440 -1.50722453409164

H -4.27117658568775 -3.79815828743115 2.03718921888751

H -5.43161658014367 -3.12419289974937 0.83920889321609

C -3.39568433038643 -2.44316488827566 0.61371520183353

C -4.41414825631966 -3.49410710127936 0.99397891293657

O -2.20028595829978 -2.63884269334579 0.96829999637758

O -3.82727507273055 -1.44544289761696 -0.05234366575017

H -4.25426993265213 -4.38134035592638 0.36737246246195

O -2.05060261961076 2.65372500875481 1.04581004938077

O -3.74970657538407 1.50914205446394 0.07920430840555

O 1.15876012000357 1.34120816675645 -2.45631766882489

V 1.86800073024669 2.30848417105988 -1.22445004793613

O 2.47695354863910 3.62905170268047 -1.89833426177844

O 0.67636675426483 2.75023239977712 -0.05364677323764

O 0.61204958528068 -2.78410188269903 0.10667886477647

V 1.78829011211649 -2.38897651046243 -1.09884110863699

O 1.10825398400812 -1.43641448893818 -2.36476574756398

O 2.36680132912552 -3.73310554558176 -1.74748494212634

O 1.44261525138785 0.36820127179132 4.42295661195838

V 1.17032305086940 0.14557076850259 2.86040915642645

O 0.25656606837496 1.53408392754534 2.28951535758339

O 0.31925268050410 -1.30144534880828 2.66093366788683

O 2.81103820092473 0.05595989925409 2.05532899187376

O 5.14754575486401 -0.04410145334295 0.76073441126475

V 3.57461214407328 -0.02136654712514 0.49395161526683

O 3.20130914395844 -1.52405844482234 -0.34370954352156

O 3.26781382541018 1.41878036331635 -0.46163879042141

H -3.79831325797349 4.23458578723248 1.90178909642892

H -4.93314367100725 3.80106435720224 0.57721598352931

C -4.28612665654133 3.41241579695259 1.37276404884321

C -3.27280265857715 2.45615610325638 0.78277140789240

H -4.92559570994344 2.85301918353812 2.06861622890138

39

zzzz-o-o

Mn -0.46947769933238 1.42410575812952 0.75109354188620

Mn -2.59929516153162 0.01371197682031 -0.81107026936459

Mn -0.04831400894730 0.00843551777546 -1.92895096546071

Mn -0.48212975415200 -1.42463961110419 0.74038232065182

O -1.60750175972034 0.00141353725573 1.14532205761763

O -1.40834785624443 1.23538180003152 -1.49004059559175

O 0.67121151122288 0.00259266443438 0.06962213740799

O -1.41172198641353 -1.21351185006184 -1.48946859121190

H -4.01642020431601 -4.18332663057927 1.72939797749555

H -5.15886995901954 -2.80379565090271 1.76290162032089

C -3.37512527850467 -2.41965326825805 0.63965823240244

C -4.44957610119601 -3.36835526908686 1.14346489489532

O -2.18051279312632 -2.64993791752345 0.96563342958089

O -3.79303883087588 -1.44886012608984 -0.07098935531539

H -5.01299331249647 -3.77604176758046 0.29444804574143

O -2.14959448579106 2.65602353707369 0.99681464985114

O -3.77676428464243 1.47998361397700 -0.04567124998249

O 1.17763828963933 1.41396276138726 -2.43315523795658

V 1.84615110463892 2.35172395570073 -1.16030472745055

O 2.46146792121726 3.68780651159049 -1.81677997979072

O 0.68237359505258 2.79093161454744 0.03817848553989

O 0.66520865771957 -2.79152729499202 0.02286078405817

V 1.83258643325123 -2.35180463100760 -1.17214882392276

O 1.17038053479113 -1.40246954123589 -2.43920306195682

O 2.44029592579323 -3.68826140373278 -1.83471527930182

O 1.62805751962880 -0.02335726229403 4.59665906485584

V 1.24359345391144 -0.01403176343131 3.01768936696845

O 0.36071029290830 1.39580632527556 2.66654348528060

O 0.35198013288338 -1.41518075047736 2.64915431004231

O 2.84847939368414 -0.01284381570835 2.07070301327829

O 5.14392726624697 -0.01328833015089 0.73589869448633

V 3.55998455477823 -0.00836870059508 0.49377984081780

O 3.25216767061752 -1.48847772174955 -0.41356495555034

O 3.26173611181184 1.47933387279006 -0.40502010761707

H -3.96905739790297 4.18232191945324 1.80513357155963

H -4.99174752017892 3.79584477138141 0.38250799699654

C -4.41344636932209 3.37617963461314 1.21538142910497

C -3.34867752474267 2.43486849820875 0.67932148833394

H -5.11154208134008 2.80283303611584 1.83952276129939

39

zzzz-o-o*

Mn -0.47092378698641 1.41133216979967 0.78239211481696

Mn -2.62960515600542 0.02124647796908 -0.79729723346845

Mn	-0.13764616815464	0.01585099911773	-2.06571862263471
Mn	-0.48983223576462	-1.41827897425266	0.75739805982017
O	-1.64189366748023	0.00004339418756	1.12828442355768
O	-1.48501762215549	1.23401024571120	-1.58261986305486
O	0.64693317584148	-0.00182012431291	0.13902271094544
O	-1.48867039896061	-1.19821392765837	-1.57748451619725
H	-3.95154176326293	-4.23517812494253	1.69527341931698
H	-5.14810212651451	-2.90004534055220	1.70614974710775
C	-3.34891544121324	-2.43868467913844	0.63780041959993
C	-4.39864880116020	-3.43090849080349	1.10532665063802
O	-2.14764673999068	-2.65514449706505	0.95574835200048
O	-3.78838409167806	-1.45570180601063	-0.03892292802387
H	-4.91836006072411	-3.85194299645352	0.23492654848968
O	-2.10517485911558	2.66188827871587	1.00569796257491
O	-3.76450760810473	1.50214337349755	-0.00383993103047
O	1.15201775331287	1.43088574074208	-2.42527561827729
V	1.83889084228906	2.35519375951753	-1.14527621061015
O	2.46803667138506	3.69068815191913	-1.79114044701067
O	0.67644600896621	2.78513334357407	0.05229901819597
O	0.65539537181672	-2.78733075897784	0.01771029736325
V	1.82031266416332	-2.35280619586332	-1.17615746008430
O	1.14119614320296	-1.40768367953055	-2.44387646750832
O	2.43823412825645	-3.68757688730047	-1.83428129488577
O	1.60811935648542	-0.04210480323691	4.63597089220584
V	1.21717623982668	-0.02844873541418	3.05796039381983
O	0.33795435407221	1.38640350180564	2.71709525351896
O	0.31434119120553	-1.42263283455017	2.69390616432106
O	2.82191386991140	-0.03848164271101	2.10583140810161
O	5.11575808758369	-0.02353357139555	0.77386391458189
V	3.53209754352894	-0.01641233274842	0.52725228526949
O	3.22700120546241	-1.48133278084099	-0.40365429465239
O	3.23737715573542	1.47157990664087	-0.37239344125230
H	-3.88126371107034	4.24541318237674	1.78947150641733
H	-4.89553263891208	3.87811535038614	0.35657743376257

C -4.34696568967489 3.44821428205496 1.20428068182263
C -3.31163917028672 2.46247679355760 0.69465729975799
H -5.07513402583020 2.90917223218579 1.82403137068430

42
z4yx-H2O-o
Mn -0.44923527700112 1.51785979742577 0.81319050200289
Mn -2.56881520743027 0.02139437217494 -0.42021546304053
Mn -0.03238728962364 0.01866925044794 -1.70543519110779
Mn -0.45695450207562 -1.49457211138876 0.80191651159097
O -1.46831347561180 0.01294328591993 1.32431557713879
O -1.29121817875631 1.26172458452688 -1.01300566019433
O 0.86374853676547 0.01362070329630 -0.18273863810108
O -1.29551024899765 -1.22288978149049 -1.01810383422736
H -4.27905178718344 -4.21092353560769 1.69509632904589
H -5.43273546019593 -2.84053755062881 1.57518711200223
C -3.48893541824682 -2.43631368152227 0.75556060692421
C -4.62779408396091 -3.38615809096732 1.06804005430986
O -2.33633447968534 -2.71909341339440 1.13712496850711
O -3.82052024247655 -1.37888254373897 0.10021116975362
H -5.04072059576377 -3.78388503478557 0.13159232727733
O -2.32868746797427 2.73945181258132 1.17692257817032
O -3.81767213582911 1.41667374466545 0.12436363426905
O 1.02130360469140 1.42911952248744 -2.38922700102050
V 1.82088962161790 2.35422909650220 -1.15080703022806
O 2.38785076300626 3.67787449627830 -1.85175565099096
O 0.72615469014758 2.84523932873929 0.08964048566246
O 0.71026835711619 -2.82427698509199 0.06817744569789
V 1.80737771196613 -2.33580947986611 -1.16966529844415
O 1.01256341420828 -1.39585211683401 -2.40001219614392
O 2.36160663170833 -3.66127138896232 -1.87736537462405
O 1.72366286808707 -0.00874938110408 4.43084851701318
V 1.38617481542344 -0.00251942563109 2.85961272139557
O 0.50657040541925 1.42423459062255 2.47337934657980

O 0.49711439371544 -1.42053540290722 2.46176635564760

O 2.96378490765778 -0.00813105012126 1.94010387754514

O 5.23389306135175 -0.00642283486949 0.53670023604324

V 3.65104459987900 -0.00108380137989 0.33421716321765

O 3.30004713826195 -1.49204500456763 -0.53251250691200

O 3.30950417570841 1.50090663042019 -0.51639737472818

H -4.28419212080915 4.16619755816762 1.85579086665758

H -5.11462351197048 3.76147204532396 0.32045934684739

C -4.63781359711198 3.35046563216312 1.21975714375854

C -3.48698040723279 2.44867863431427 0.81937794684824

H -5.39396420296314 2.75999629391312 1.75171737542401

O -1.02903270003613 0.00342376835483 -3.55889994736934

H -0.66779731828580 -0.76678063984049 -4.02586452585779

H -0.70395398750949 0.77595910637441 -4.04811950634054

42

xzxx-H2O-o*

Mn -0.32083016443143 1.44708343422511 0.53329491008925

Mn -2.41188015302310 -0.77534723470076 0.62852931019932

Mn -0.07856772921585 -0.73249640831797 -1.29881094588520

Mn 0.20743331807548 -1.39510027384004 1.53757155613060

O -1.28661836659808 -0.39223466904169 2.00855912941124

O -1.34898389934544 0.55683627555663 -0.81065651951810

O 0.99377010780441 0.11281379229769 0.29354607208840

O -0.97046711976422 -1.88598923216879 0.04095098453547

H -5.97461477482919 -3.95332312435491 0.41170055573621

H -6.11023802144244 -2.86952842044711 -1.01686749272555

C -4.39889162766746 -2.48177780460466 0.23745302316863

C -5.42055669485371 -3.42223498573757 -0.36846828447392

O -4.08993125905704 -2.54672514456420 1.44340842933217

O -3.84125832973047 -1.63369355063694 -0.55729163573526

H -4.89438596907492 -4.15486630609496 -0.99583488569380

O -2.00405499444789 2.41873380701820 1.11911752127516

O -3.58108710209058 0.79985261934200 1.09719959418790

O 0.69096525882864 0.41342583315749 -2.62851053350248

V 1.52641964711249 1.83232556146406 -2.02202338517312

O 1.87601456317643 2.81419976487205 -3.25002791044318

O 0.59072963253337 2.70326896518328 -0.93044085244133

O 1.47080492786248 -2.74822686021307 1.06147782914961

V 2.26319732315611 -2.61347770366551 -0.45364148358492

O 1.15668066286212 -2.14103057199668 -1.68310747321091

O 2.91051611394195 -4.03889193994496 -0.82429351239049

O 2.41772805393382 1.54323982655778 3.95528108447292

V 1.86300098247928 0.92826568309273 2.58333123561916

O 0.65302347260456 2.05365341424090 2.04866878433164

O 1.26868291785746 -0.66024979352300 2.94780981486145

O 3.39645061167600 0.86976930711559 1.57475035587410

O 5.39956765522368 0.55959034828842 -0.12501508001468

V 3.81611538134355 0.34847688978981 -0.04260216776197

O 3.59160864792471 -1.37615434483215 -0.31965531269476

O 3.15305443923663 1.33698413325881 -1.31882703418963

H -3.78364429689971 3.81668975497722 2.24758749214288

H -4.66847030799233 3.43873233966345 0.75334315451357

C -4.23593041011597 3.00712540717579 1.66703878711078

C -3.18412507189579 1.99281161642871 1.26985154405850

H -5.03852016641821 2.52496184139689 2.23311158428592

O -1.73708873128704 1.69184534481010 -3.25021503502000

H -0.87943833343154 1.30439513012963 -3.48537307253724

H -1.75586419402050 1.40966827864265 -2.30697513557835

42

4z44-o-H2O

Mn -0.51208528550141 1.45985370476006 0.59295609064296

Mn -2.57250585411240 0.01605540306527 -0.63141889370221

Mn -0.00383426280029 0.00177097345231 -1.95567708155905

Mn -0.53315836766549 -1.46374169170707 0.58432029770917

O -1.72638858938731 0.00559485522452 0.96121662329710

O -1.30423482726903 1.24126280651433 -1.19757185557030

O	0.59337365317870	-0.00995690994334	0.15498387417611
O	-1.31966913639566	-1.22465542267339	-1.20348143601996
H	-3.86243677770054	-4.18595784316684	1.78059758014357
H	-4.97247114290923	-2.78276632861763	1.92170568357016
C	-3.27053177689238	-2.41380452738658	0.71228593042567
C	-4.31855791747323	-3.34998900464784	1.24635222441480
O	-2.05262464542456	-2.65344534075140	0.97065937931084
O	-3.69325816101798	-1.41473535826208	0.03747138136295
H	-4.93843521109968	-3.71478265399243	0.41847043664791
O	-2.00797832168970	2.66991753557967	0.98832580293102
O	-3.66839061193734	1.45991975810711	0.05176331761497
O	1.16858104835179	1.35937574042383	-2.41706089297260
V	1.89844396641557	2.32088448346343	-1.17933223494493
O	2.49469033538273	3.63550124599001	-1.83382577578940
O	0.66866209665740	2.71959384528422	0.01122869750532
O	0.63596813893363	-2.73253963593903	-0.00105363620134
V	1.87029196764235	-2.34002603812142	-1.18892514181786
O	1.15053874923890	-1.36819740557514	-2.42468144876019
O	2.44940787921464	-3.66015541585591	-1.84659557324558
O	1.07863511221996	-0.02441846525239	4.34366737984172
V	0.98211513306405	-0.01933852053041	2.76601944663954
O	0.09661675889280	1.42659904203015	2.30656697538175
O	0.07583841935183	-1.44906606408812	2.29867283811149
O	2.65915801721992	-0.02903654454760	2.11759187542008
O	5.07570131127721	-0.03058539472460	0.94018538625278
V	3.53391839264644	-0.02321612671888	0.58611927338102
O	3.21755425948744	-1.46156825012556	-0.37510165119748
O	3.23195818437867	1.42795786572041	-0.36126720309025
H	-3.79304184291486	4.23438856350169	1.79186926322015
H	-4.90442511315403	3.74679203243162	0.46299947390089
C	-4.26313600364812	3.39287900098739	1.27883782021242
C	-3.23009693895513	2.44810632168428	0.73193178356423
H	-4.89877391393869	2.83419238386210	1.97863798234235
O	-3.62945409068587	0.02143697775395	-2.32676109502981

H -3.59911838296868 -0.76729855625788 -2.89255320987747

H -3.58447824801229 0.80132095904919 -2.90369668824255

42

4z4x-o-H2O

Mn -0.44963385699820 1.43651001919023 0.64845501391798

Mn -2.53202514630909 -0.05452984951122 -0.53792378266917

Mn -0.05391852057093 -0.04118901920126 -1.91514506440067

Mn -0.46768104514487 -1.57795038122689 0.79169126109622

O -1.64933508055512 -0.08162921886784 1.06302819618970

O -1.30160913107487 1.21794102885464 -1.12830481327430

O 0.77630589418511 0.14260661642854 0.22189643136822

O -1.37950883649117 -1.25890362343208 -1.29559869048238

H -4.06834003246605 -4.41340981721537 1.44806520262909

H -5.14718381848312 -3.00194562087922 1.75550649400314

C -3.36515266036080 -2.52706547958760 0.66005622259796

C -4.47801737741719 -3.48641168347537 1.02131337879396

O -2.17564363170706 -2.80981807341253 0.96911659965081

O -3.74145624595729 -1.45040255801650 0.04797227292898

H -5.08059472818410 -3.71137959404021 0.12451363062917

O -2.02604650609867 2.63000437682074 1.08233018199402

O -3.68277632875489 1.38643649486187 0.14479386395579

O 1.06496006934510 1.34627684815354 -2.47415701940693

V 1.77454750167166 2.33401251182912 -1.21279515069844

O 2.30426334872057 3.69524548447723 -1.90275138618788

O 0.58333069995761 2.81722534026367 0.00134297273758

O 0.69177860784996 -2.84654118066686 0.11789312430084

V 1.84342266808401 -2.36593265551111 -1.12037640202406

O 1.08903554170281 -1.43291503412267 -2.39557552417767

O 2.46263137849872 -3.70929936605540 -1.77225253807830

O 1.32616558549502 0.18091723059871 4.42538616810845

V 1.14237132475965 0.08378506679019 2.82234011031659

O 0.16354044984072 1.50083545668411 2.37717075997175

O 0.30866343128650 -1.41646591209693 2.50750527304620

O 2.84614967027614 0.12502377744603 2.15393364110240

O 5.11131337307830 0.07981938500820 0.74646909040992

V 3.51598196665884 0.08390132597671 0.51242673115457

O 3.20220286761236 -1.42703843499865 -0.36901420908305

O 3.23070147389180 1.54779262219816 -0.45608974997594

H -3.80431255875884 4.32252309065667 1.62080892760026

H -4.97949567745259 3.62510317930457 0.43217203357333

C -4.28111952810186 3.40110941880161 1.25573305049885

C -3.24241537574769 2.40719865407397 0.79216695667477

H -4.86706535489291 2.94335842817360 2.07396986117380

O -3.62340205381071 0.07642527506384 -2.28317717444886

H -3.48560452367682 0.93547721363884 -2.73172114713041

H -3.33265983389996 -0.60257934297709 -2.92674179838630

42

zzy4-o-H2O

Mn -0.47230008104030 1.54797534202031 0.70150230197412

Mn -2.60282959723254 0.11782040588445 -0.68385909900207

Mn 0.02948043327632 0.07670878553795 -1.85147293242378

Mn -0.48832121768385 -1.37491722673531 0.58925143934951

O -1.53591384658391 0.05784075428164 1.14025413608594

O -1.23790489744782 1.34196483109288 -1.17639661821282

O 0.79414172368513 -0.19379985297072 0.08924273734315

O -1.29304590639427 -1.15364596451743 -1.18781047621151

H -3.85349136238153 -4.11919460401915 1.85443534018311

H -4.97968231240284 -2.73261300319754 1.99534578334445

C -3.28359621843221 -2.34146837852097 0.75920523860694

C -4.32246998824081 -3.29147825779782 1.31637178892074

O -2.06942202322592 -2.57970932738692 1.01913808407204

O -3.72532359943844 -1.36216911382378 0.08123864696107

H -4.94601771902778 -3.67622899840397 0.50007786637841

O -2.31311875418156 2.76059947647599 1.09583666717311

O -3.81978137332036 1.52018168626669 -0.03154492002876

O 1.26150953339047 1.43545306121010 -2.34163720436241

V 1.97410191301475 2.38957019257469 -1.09267010446405

O 2.64004138878217 3.69585830820629 -1.74823162026040

O 0.79413952976862 2.84498421450870 0.07648466019798

O 0.58730814652552 -2.81665790392069 -0.07483611908972

V 1.79283774408356 -2.32448456820578 -1.21678622023594

O 1.10535852454512 -1.35925279580739 -2.47850047543151

O 2.38145414405449 -3.64777098304085 -1.90245796991774

O 1.32338376882702 -0.16921258855090 4.37055314938269

V 1.15643247365416 -0.09248789208490 2.77939708468115

O 0.39215356541945 1.40517124187711 2.41808518463040

O 0.23865776213996 -1.49818105454995 2.30377220673185

O 2.84941386474116 -0.17480555219477 2.10323437429290

O 5.16131316211468 -0.13476474194073 0.78621465171233

V 3.58874133064133 -0.09951385105392 0.51665437666791

O 3.26027550727099 -1.51028281895020 -0.46944475993845

O 3.30153835759282 1.43977145179900 -0.29138846959020

H -4.24513513548298 4.07714756328238 1.98498035982966

H -5.19669487680025 3.75269229102933 0.50041196190506

C -4.62531104896516 3.29771713383382 1.31901740384560

C -3.48051260555150 2.47591321981956 0.75864146391331

H -5.30731846205350 2.63816866448164 1.87086458842631

O -3.70274636176507 -0.04380969775251 -2.67276686213694

H -3.55241557943022 0.72338505805230 -3.24339469311123

H -3.35656190644481 -0.79835250680866 -3.17057995219224

42

xzxx-o-H2O

Mn -0.39352251699880 1.63493582981755 0.58686688678603

Mn -2.66092823991366 -0.00654633081161 -0.49674433909671

Mn 0.10869981117644 0.19211225007915 -1.77320500485800

Mn -0.54086357775239 -1.49703373105525 0.57371693890465

O -1.83186152999829 -0.20204293433305 1.06609993750506

O -1.10253452737718 1.47160641575749 -1.17908950852575

O 0.80586429118629 0.24414831570405 0.19639715752483

O	-1.23093301687698	-1.10915449669282	-1.23309985391878
H	-4.19577169339349	-4.58895275003078	1.65150116020542
H	-5.27229562354811	-3.16900122971163	1.82136300513883
C	-3.58061436919759	-2.80964230732375	0.54317707243065
C	-4.64520566688969	-3.74188978743499	1.12461162230675
O	-2.37491162146119	-3.05822212325437	0.77290054990829
O	-4.03387667357198	-1.82299740495765	-0.12380902020765
H	-5.30402665157149	-4.10590552661507	0.32572072507803
O	-2.01356687630659	2.80282498707532	1.06732037244602
O	-3.72337462642808	1.59844059366163	0.21194832427348
O	1.33917039754573	1.53237759558759	-2.38126051803785
V	2.10739562663509	2.52736741481295	-1.18799161622173
O	2.82942651421016	3.77291208015086	-1.93555648959885
O	1.02416690373677	3.11966299456817	-0.04437517355927
O	0.76227980136083	-2.76777338022423	-0.05575618233396
V	1.95098713028168	-2.27603928631192	-1.19989543477354
O	1.27283154670424	-1.25071348835364	-2.38879944263560
O	2.58458039891388	-3.58035964411065	-1.91287862504002
O	1.24755009509620	-0.00624948855407	4.29893266043628
V	1.04311041572466	0.03775647709375	2.70238185661994
O	0.17313019506382	1.50628873125400	2.42229814928766
O	0.20546786210329	-1.43779826464081	2.33130435161558
O	2.78952945850700	0.08086053331112	2.10401169916398
O	5.15568812970441	0.02114489151036	0.92483161286896
V	3.59668358260194	0.07898434076976	0.55495605803317
O	3.31995507713195	-1.39719619168642	-0.37603180308749
O	3.44375459986357	1.56422929271286	-0.35818175469775
H	-3.71601772045859	4.49347865456415	1.75379341924504
H	-4.68320768173443	4.11142793201605	0.29678057186021
C	-4.21527862854084	3.69475815806409	1.19870600043294
C	-3.23022022001307	2.61254465788380	0.79634899323098
H	-5.01422066389404	3.25518007493317	1.80763583316674
O	-3.95285328919953	-0.13558858996456	-2.20330625687477
H	-4.34391572997319	-0.99463434408991	-1.95435622406255

H -3.47790369244876 -0.29117692117072 -3.03283471093929

42

xzzx-o-H2O*

Mn -0.49289736429421 1.44689923370195 0.67720221711266

Mn -2.77398722091137 -0.17154525107908 -0.67968674466484

Mn 0.00866838103807 0.07485961148035 -2.13478007223338

Mn -0.51504300853557 -1.52125828980869 0.41193384725490

O -1.70624691075045 -0.06026090079615 0.84333771271743

O -1.36136353589806 1.36794521237845 -2.20801626383055

O 0.73196852072664 0.28488311850814 -0.01615142008941

O -1.24991139712536 -1.18086376086467 -1.39261130091136

H -3.84246359516270 -4.35949600621431 2.15345023706268

H -5.00994295890931 -3.00096735209255 2.24184578819451

C -3.41966791510547 -2.69150710609734 0.82390230598359

C -4.38252073890150 -3.60497306335450 1.57399960591374

O -2.19249399924751 -2.95755232925061 0.85756381609229

O -3.94740682212991 -1.71274916946039 0.20987202127213

H -5.05230470128308 -4.10048252809342 0.85891987505199

O -2.10223361192449 2.65670085883409 0.92195622918063

O -3.76677753813168 1.47128233268929 -0.03039713671716

O 1.30020956512272 1.45179777301162 -2.45870771716860

V 1.84286100264073 2.34651419093445 -1.07293366015022

O 2.50999254121985 3.67508545562837 -1.68448454417634

O 0.59013945257433 2.87860988139257 0.00950818320891

O 0.76084413860057 -2.82420367027014 -0.20621291878777

V 1.97794158813415 -2.31319739087061 -1.30017591938616

O 1.28543304093555 -1.33326649200846 -2.53151794370781

O 2.68932830739240 -3.59737126155659 -1.96981845040603

O 1.10474886966222 -0.48924758152443 4.46021659681718

V 0.96235312785793 -0.21205559526587 2.87650467436851

O 0.10666641408135 1.23218634762625 2.65995301809706

O 0.17411110527983 -1.58536469539817 2.17503673820975

O 2.65976444043026 -0.03882291075247 2.19027529998082

O 5.02878289776636 0.01955936149958 1.01465522450230

V 3.47139690274446 0.04863789983505 0.64320301841383

O 3.22439343626676 -1.36088207257540 -0.37910070545746

O 3.27300353094865 1.56164831129021 -0.22317184195473

H -3.84624930926014 4.30174426045457 1.62218466580792

H -4.81587745534194 3.92176009733942 0.16340832048196

C -4.32387819483061 3.50194595277652 1.05034979079866

C -3.31136470590674 2.46190461415962 0.61645172626596

H -5.09968657082977 3.01775013362204 1.65610796405346

O -3.69466040773964 -0.08228473781309 -2.31843811426287

H -4.38373688797825 0.59105815863081 -2.24165548634700

H -2.18952441322489 0.98970135935355 -2.56754563659123

41

zzyx-OH-o*

Mn -0.17934383360211 1.40822494360796 0.80888925782039

Mn -2.39174526396286 -0.03235334313611 -0.63243121138925

Mn 0.09485624902653 -0.04255013335568 -1.84969633214483

Mn -0.18649946787847 -1.47471467806780 0.81311546688733

O -1.23993078042308 -0.03089049182953 1.36884663094617

O -1.03350187242670 1.24098117492377 -0.89760078878958

O 1.31731990048700 -0.03550744259109 0.13023212736907

O -1.03569524941776 -1.30235345385259 -0.90185384750768

H -4.44895453326551 -4.09884391827629 1.65429870470264

H -5.46933018664358 -2.63984939638935 1.45749912753183

C -3.47252936065542 -2.42757885021757 0.67367754606314

C -4.70532704029499 -3.27164485010404 0.98618171983986

O -2.38612205596524 -2.72095142436723 1.19282220837904

O -3.70588166598233 -1.44170183666420 -0.12781810167813

H -5.13657344444684 -3.66610418136720 0.05697664818052

O -2.38043083274508 2.67378904472980 1.16931605195138

O -3.70068728901829 1.38331037136906 -0.14040447568625

O 1.21674185216186 1.39748505726597 -2.45541566502246

V 1.94263105540183 2.36455499984579 -1.21270639992319

O 2.53528810021423 3.69435044035654 -1.92266392662655

O 0.86585059453545 2.86325450332876 0.04092508676841

O 0.85143195120155 -2.93706356341598 0.04901914615765

V 1.93779404378626 -2.44846582663640 -1.20108600711346

O 1.21681450426821 -1.48769389886079 -2.44941042313921

O 2.52988519964833 -3.78239808910749 -1.90329659655734

O 1.74563891899969 -0.03200569405817 4.53561439518838

V 1.46818565429407 -0.03290058324954 2.94008570941205

O 0.65575201818686 1.44711118484132 2.55241540487078

O 0.65008739290743 -1.51100884772578 2.55491454731566

O 3.08593196166658 -0.03636122210335 2.15651318250541

O 5.02363822706165 -0.03915180799023 0.54712008527598

V 3.41191863817168 -0.03880784178482 0.38047667022055

O 3.37507835078194 -1.61196635601938 -0.50853504723551

O 3.37938627467654 1.53072704038822 -0.51711802313864

H -4.40171053280112 4.14555389618937 1.49121509225321

H -5.03745828225282 3.67442793386767 -0.11672294719043

C -4.66527289159283 3.30203595619583 0.84660334535964

C -3.45742943623437 2.39690655794815 0.62306689984434

H -5.47629141909575 2.72342257025542 1.30727293588769

O -1.73262594491441 0.00783173156446 -3.06916829819053

H -1.81755550385805 0.96476132449251 -3.16662589939817

41

zxyx-OH-o*

Mn -0.27979503285383 1.29286464899864 0.74034783653955

Mn -2.36034002290598 -0.49132108887360 -0.23298660300718

Mn 0.20287546448742 -0.06714072228150 -1.89192462784675

Mn 0.14158424649883 -1.54271104215702 0.76265518767488

O -1.08327208294602 -0.26740164158734 1.43897188610775

O -1.23631575177851 0.85508755716429 -0.96029040584128

O 1.15537023147757 -0.01121571957292 -0.24980934169816

O -1.13598846855380 -1.80487964230462 -0.59972050907608

H -5.99918614183666 -3.61197792949887 1.48261733787154

H	-6.50082497572072	-1.99182838024342	0.94968632146465
C	-4.38258064085352	-2.31122404035838	0.84895893860777
C	-5.81691798368276	-2.82711723574975	0.74208984245498
O	-3.74835755361962	-2.47422356841282	1.89466895385965
O	-3.95843975252159	-1.68692648955754	-0.20016070552786
H	-6.03726648112739	-3.19679785104606	-0.26575804962370
O	-2.22277555398198	2.43563661260215	1.29377800920916
O	-3.65580782019907	0.95258324422422	0.37942099013787
O	1.16849274205466	1.82239480981162	-2.53311304842055
V	1.76181525868738	2.65682293132422	-1.20969784420374
O	2.20790651823019	4.13944347371280	-1.69428138836761
O	0.55875572550897	2.84802127651411	0.02720033672538
O	1.41520186431112	-2.80273635778184	0.02227127331914
V	2.36960226869668	-2.17704029867830	-1.27512388369238
O	1.47803915531627	-1.41369336542871	-2.52326259475531
O	3.10061643687550	-3.44847382126515	-1.95731969877049
O	2.20301029865043	0.14313833300520	4.36809078512970
V	1.83305494524063	0.09405368392858	2.79202112803637
O	0.76786102348219	1.37498531145667	2.41268290813808
O	1.15209871007808	-1.43461887019566	2.42860474714320
O	3.39768277332953	0.27731948353100	1.85904268125234
O	5.47242156366906	0.63227925130555	0.24962768529595
V	3.88585997252765	0.40511885572164	0.17314247156953
O	3.75520503213026	-1.10836968544220	-0.71709625310054
O	3.31089963812568	1.86117629399369	-0.59374993425994
H	-4.24998496020396	3.73209032053383	2.01703087030948
H	-5.00115171630149	3.33613404878311	0.44592244288612
C	-4.55936011838059	2.91448933024277	1.35922916557547
C	-3.36608692698538	2.03922212540060	0.98910615707740
H	-5.33242410645169	2.30995944343978	1.84863645114786
O	-0.80143760805487	-0.30948598030854	-3.47376098178936
H	-0.36670617041866	-1.06777430495020	-3.88320153755289

4444-OH-H2O

Mn	-0.54905076640874	1.43337114676905	0.57632114683640
Mn	-2.61467177574861	-0.03791821068756	-0.58127275851554
Mn	-0.08792062543516	-0.04385557298025	-1.94292939087469
Mn	-0.54297827178311	-1.49497592671924	0.59170869876762
O	-1.72620041602882	-0.02905375938647	1.00331537014421
O	-1.35480992384055	1.20728925155559	-1.14709611044231
O	0.54271378793401	-0.03190427475041	-0.02014394230955
O	-1.35902860532119	-1.28194341504313	-1.14826526786103
H	-3.79700115612746	-4.25978289280611	1.83186168821238
H	-4.99465860078236	-2.91950621551038	1.88775604512439
C	-3.24816246457189	-2.47315495673322	0.76984891069682
C	-4.26938901017902	-3.45636818594523	1.26350671572624
O	-2.02286036166634	-2.68657069529424	1.03158907665116
O	-3.69474397829359	-1.47236536546455	0.11448783259702
H	-4.81429190584088	-3.86608852691444	0.40351215951941
O	-2.04010651719166	2.62770131100907	0.99588191489393
O	-3.70160888020550	1.40179090944736	0.07493460704239
O	1.06737507132331	1.29060834817888	-2.39839502192851
V	1.84108452994449	2.28170871608012	-1.19061454611767
O	2.40208789437151	3.59087371966082	-1.87863707425459
O	0.61779012479777	2.70448574964055	-0.01337103899120
O	0.62547447056470	-2.76918726692612	0.02257645642878
V	1.86332581183273	-2.35991425008168	-1.14823931640011
O	1.11024682704720	-1.37010115141743	-2.35767771362428
O	2.42744367839832	-3.67164582284175	-1.83135299149952
O	1.11319599534210	-0.00397941152541	4.36734166791861
V	1.01987627350569	-0.01041991754050	2.78853093552295
O	0.13896395076940	1.40087324955281	2.26102916167797
O	0.14306680539867	-1.43188235826128	2.27304161078295
O	2.66602897749017	-0.00403090473075	2.07909714209930
O	5.09084607387590	-0.01068510061203	0.90363819253784
V	3.54884841934341	-0.02487427848558	0.55662111788623
O	3.22964260562305	-1.49764857250472	-0.35081212045243

O 3.20541016479055 1.41008865391203 -0.40596639366087

H -3.82479884056217 4.22343750254227 1.73641871732997

H -4.82110551927389 3.80058219219468 0.30125309978326

C -4.28840646868869 3.40866985019269 1.17715499641721

C -3.26060131322087 2.41571408088200 0.71856859668558

H -5.02291515646132 2.88409925009887 1.80112429035608

O -1.03854058395339 -0.05939550643143 -3.49825001379049

O -3.44448662260075 -0.05225106897266 -2.37022155717421

H -0.82310041282824 0.72063625249016 -4.03307067134636

H -4.00757540745975 0.70916293276508 -2.58704223565466

H -2.66250187787894 -0.04713450840549 -3.00777498674067

44

xxyx-H2O-OH

Mn -0.37669935776638 1.49935608657810 0.50008820425853

Mn -2.64909066632351 -0.09783463389351 -0.61845138434324

Mn 0.17128135463919 0.05603540579686 -1.89940959432216

Mn -0.52706472861952 -1.52711276453749 0.56227351998423

O -1.59952061454623 0.03962204962103 0.96410912924698

O -0.98595028098735 1.37943465256956 -1.25137014949686

O 1.00627644634253 -0.08152384660112 -0.24041027311354

O -1.41015627446732 -1.40846155168159 -1.09195001921297

H -4.06595106755380 -4.53159938035136 2.10512721647248

H -5.31759702393534 -3.24717233695352 2.13718690706698

C -3.52963284476421 -2.68708798724938 1.05106474541021

C -4.54599843300609 -3.73504586509469 1.52770970033823

O -2.31270645999478 -2.90672044161334 1.29119384597098

O -4.02184584749746 -1.69041519467585 0.45082132457263

H -5.05279887909159 -4.17244056956056 0.65614649002683

O -2.33875141776190 2.81043665940266 0.93902219928959

O -3.84522111964300 1.44669739237941 -0.04801851745323

O 1.52060238561375 1.69984910607243 -2.54230430456537

V 2.11754196281136 2.47292198320060 -1.17282720581737

O 2.82567900478714 3.86716125586876 -1.61716113278718

O 0.91352422362707 2.85828068136636 0.01294762357771
O 0.72977138318561 -2.87884204299518 -0.06559807389093
V 1.88600843923403 -2.33610387493859 -1.22599981623506
O 1.20410984445887 -1.45482009441252 -2.54331809386977
O 2.53218176866504 -3.66097769849229 -1.89278874053321
O 1.33101084335534 -0.04142113188250 4.37949969958514
V 1.12403712474885 -0.03185656118520 2.77278613528548
O 0.30736376011881 1.40769874518701 2.33621600465636
O 0.28124172346824 -1.45523181215088 2.32594040520240
O 2.77169330636004 -0.04613430889713 2.00874194749007
O 5.12476702581969 -0.10777743994762 0.84890722044101
V 3.57030825851706 -0.05215273963923 0.44222460985966
O 3.37558284034680 -1.53292492517816 -0.48912470897123
O 3.47820265921476 1.45751835598627 -0.44181224505837
H -4.28662365016122 4.23477677801355 1.62828690290442
H -5.07460802255622 3.86475214526059 0.06660339547336
C -4.64132048228239 3.42990722766945 0.97760263880809
C -3.49483761995857 2.49425335045085 0.60280397827308
H -5.43544480961161 2.86379780426240 1.47991168514248
O -1.08901164992074 0.13744599814764 -3.62240504383591
O -3.40443728106141 -0.07777430799945 -2.41736075321744
H -1.06357769961320 1.08853017904470 -3.79965086661481
H -2.01308696854836 0.00959033897172 -3.19224267704812
H -3.80160515564188 0.79666031408113 -2.52530692895014

44

yzzx-H2O-OH*

Mn -0.48316533103738 1.54206946108154 0.54829997433681
Mn -2.61914853205581 0.06866189165634 -0.88749599012886
Mn 0.12262345319875 0.08950209997176 -1.95898712164214
Mn -0.45177249201243 -1.44060394501131 0.43343251590232
O -1.65183830738843 0.03598420377743 0.76146775100003
O -1.30837403618448 1.23336547765488 -1.60829647882643
O 0.84527592200119 0.34672409122044 0.00871613168414

O	-0.98742303846411	-1.32397269611963	-1.38054225534545
H	-4.23866159760860	-4.14289055162556	1.59155444947296
H	-5.39893791552376	-2.78223276466945	1.44389341912076
C	-3.48835373673335	-2.43379836369123	0.48971793599909
C	-4.61988403834679	-3.35881072837073	0.93018775327827
O	-2.32220166698902	-2.74853837825230	0.80681057456669
O	-3.84316087035539	-1.40685893355134	-0.18204059289507
H	-5.08110264253423	-3.82404050126802	0.04851796834458
O	-1.99519246223225	2.81638826807861	1.03424508818341
O	-3.77992740152659	1.80769192039921	0.10237149054628
O	1.26768744885023	1.56699827671538	-2.48385323877686
V	1.87916356222854	2.46086783550399	-1.13915993493111
O	2.54078719846407	3.78866972292462	-1.77809095328901
O	0.67246636883445	2.99181246571952	-0.02243248027423
O	0.89223811458831	-2.75657533719276	-0.09785310742881
V	2.09831235938515	-2.22958894981363	-1.19651527416854
O	1.47510128737570	-1.24960361334380	-2.45515563963312
O	2.83118044396213	-3.51837067393054	-1.84961813984481
O	1.15010772232924	-0.25326222626308	4.47048098336873
V	0.98099625279713	-0.02773929131883	2.87379679769986
O	0.11207290214251	1.38748697444788	2.63727408858834
O	0.18893206255031	-1.43422139591671	2.23768752714013
O	2.69453908385931	0.17724666868266	2.20814499386946
O	5.05880819146505	0.23591571786483	1.06107894034407
V	3.50368600728932	0.20994488443994	0.66144857198299
O	3.34244171780932	-1.28197109172752	-0.25894058556730
O	3.32021040748838	1.67111791899288	-0.30208888816538
H	-3.63248705501948	4.22788572284076	2.32097163829031
H	-4.93454686910814	3.95937026741138	1.11380075800224
C	-4.17857733501198	3.47534968675006	1.74473155067453
C	-3.24733510308029	2.62798600608928	0.88043019944096
H	-4.71326844299123	2.80899869458945	2.43597625045198
O	-1.82567267403078	-1.51701157157974	-3.86904458964190
O	-3.61116938742921	0.12962865659165	-2.51651557094590

H -1.41822325331568 -1.71872121473894 -2.99621377666328

H -2.56073629135174 -0.94849190695897 -3.54730340938561

H -3.19782402628774 0.89093222193963 -2.94518432473509

43

4xxx-OH-OH*

Mn -0.45187921797894 1.49983605375326 0.49826364274169

Mn -2.58615843538963 -0.14454221078805 -0.54860658444081

Mn 0.05252286740709 0.09463922071956 -1.97999812338155

Mn -0.50688566110559 -1.53862598013579 0.60941163207308

O -1.75261867112252 -0.22677571600173 1.08813486809028

O -1.40159912233259 1.19162182935940 -1.11537788345187

O 0.73527112678043 0.17305209729478 -0.16433579276628

O -1.40926180738354 -1.42004123567414 -1.05620597363502

H -4.67625937247042 -4.14581185676581 1.82777584852554

H -5.64407704016926 -2.64772413971593 1.63795911944628

C -3.67540823388591 -2.54763535669674 0.74793143661812

C -4.92905144923678 -3.32344485042309 1.15203645747807

O -2.56042376907525 -2.96489724152006 1.09786974693257

O -3.92164456410478 -1.49671705046106 0.04745482360426

H -5.41881576266876 -3.72735775518582 0.25575780992531

O -2.06628461770792 2.64762295094847 1.09416601400395

O -3.73620378557102 1.37872046883196 0.24350410617782

O 1.29761777550560 1.71487043973605 -2.54340597011133

V 1.99256727162555 2.59727000865295 -1.28060931016992

O 2.71205946653253 3.91995037063028 -1.90999386308306

O 0.82049807567666 3.07734828700999 -0.14809621339410

O 0.80969277140351 -2.80384150519290 -0.02305023439065

V 1.99091026602285 -2.31372563638878 -1.18682175677830

O 1.31900233054526 -1.38592159540678 -2.43603452957522

O 2.66565140626023 -3.63157376513676 -1.84330032481748

O 1.35526753084795 0.08771604486868 4.35005447297550

V 1.15022186870933 0.05601573979405 2.74791598831469

O 0.28972067875820 1.46309599275471 2.28663185153949

O	0.34821143984732	-1.40851050501985	2.34205411164852
O	2.80481098057655	0.12859721014954	1.99102122157889
O	5.18605292402295	0.15055910208238	0.79676891126732
V	3.62056758427133	0.14200419546604	0.43743326636322
O	3.37222208706458	-1.37398534391699	-0.42242517173303
O	3.36267279178389	1.59580887630057	-0.48118431254763
H	-3.85060271564369	4.14202588558575	2.00376858046858
H	-4.87980015631712	3.79117565099207	0.58086795563336
C	-4.32043221547675	3.34873977653727	1.41564449644421
C	-3.28201476449860	2.37861162595802	0.87246182104968
H	-5.03981723089633	2.79944893383300	2.03588925350589
O	-0.88627158445697	-0.01131201778616	-3.57530651497186
O	-3.49513856143564	-0.09749768853554	-2.21227876745124
H	-1.81141434752224	-0.01233257101715	-3.25994358540977
H	-3.70120115719147	-1.02325773948962	-2.39406052429727

43

4xyx-OH-OH

Mn	-0.46187964476005	1.39437644475111	0.58774120333477
Mn	-2.53343891291204	-0.10760577651366	-0.60960359185701
Mn	0.11983727871535	0.01602879610217	-2.03417262631148
Mn	-0.42512299387788	-1.57289902994741	0.51371350777233
O	-1.58703934850662	-0.10628411633024	1.01742442368650
O	-1.32288828632981	1.18682234534356	-1.12568632692373
O	0.92700014327020	-0.05090583964102	-0.31834412042461
O	-1.38289385166765	-1.41402850264821	-1.11846790070038
H	-4.39990810928664	-4.00046400658668	2.12732736041101
H	-5.37908419424976	-2.50710559794599	1.96300367612183
C	-3.50904726196968	-2.45589480633134	0.88257647832592
C	-4.71536173078830	-3.20872934426884	1.44187118690260
O	-2.36239619658704	-2.84185604730648	1.17806465602480
O	-3.81655162413843	-1.46214289148324	0.13352506870870
H	-5.29095024688282	-3.64835407567563	0.61647655041800
O	-2.34190413807792	2.68801888659176	1.14785167055751

O	-3.79824464704277	1.33228090280394	0.08690080159500
O	1.27622176722126	1.73775765616289	-2.48785423866809
V	1.90794845351273	2.47568193768236	-1.11901585059799
O	2.52590347865967	3.90390467482237	-1.56463511889254
O	0.68553604833708	2.81319624548693	0.07180724378526
O	0.79135793964603	-2.89386953028731	-0.13604453790334
V	1.98282206276249	-2.34337790091058	-1.27937212785086
O	1.33035418526724	-1.48011762310648	-2.59002108665911
O	2.66914305144155	-3.66563408587180	-1.91099358526289
O	1.40540706411544	-0.14779522113969	4.36475013635500
V	1.18286608822473	-0.10097219641756	2.76408920993228
O	0.28860882254418	1.30617675372296	2.37424533573924
O	0.38774208964168	-1.53575579716620	2.26354720024984
O	2.80919587045985	-0.00668068561064	1.96741506689588
O	5.14997337056215	0.04277502753239	0.75559868376721
V	3.58609600262296	0.02979553695693	0.39103111284165
O	3.41542268461839	-1.46450732267648	-0.51717321544969
O	3.36872309376493	1.54503052212548	-0.46366052521798
H	-4.34253322040779	4.02867090888629	1.87611642285688
H	-5.12836435297717	3.67579819860904	0.30958025286750
C	-4.66903568288813	3.23077700881334	1.20263597189858
C	-3.48895574323323	2.35453632469368	0.78855711818120
H	-5.43630986014134	2.61847710250318	1.69262677509441
O	-0.83452915121136	-0.00573380839290	-3.64113264784532
O	-3.45098064959481	-0.04434985668425	-2.24539193944667
H	-1.75366204851995	-0.08091175654639	-3.32834744782623
H	-3.49680059933670	0.89906954589864	-2.44881822648601

41

4444-O-O

Mn	-0.59430139910664	1.39732060631989	0.51474941196223
Mn	-2.71163462401892	0.01073216484134	-0.82446095522972
Mn	-0.11166960639724	0.01539979323057	-2.07217936744747
Mn	-0.60168401849769	-1.40842042302390	0.49060240288133

O	-1.72334016471752	-0.00589756800175	0.97258361583845
O	-1.34814095769169	1.26983600143218	-1.20634028938217
O	0.54941689536349	-0.00540136445306	0.01373249962571
O	-1.35570842238458	-1.24788223616977	-1.22946432418784
H	-3.77357297758032	-4.25320090680842	1.83672436456060
H	-5.00930116425905	-2.95258677768692	1.87961386127930
C	-3.29165032990600	-2.46489366499441	0.70538442387149
C	-4.27602069038978	-3.47718666702446	1.25415435223218
O	-2.05441992974407	-2.67193565061720	0.94325704261244
O	-3.77686956051124	-1.50193191158681	0.05704715252088
H	-4.82530067431620	-3.93268228429053	0.42006851393463
O	-2.03445674829869	2.66480867118482	0.98730266900348
O	-3.76521324542245	1.52542540495626	0.07804285231258
O	1.19777636361803	1.39123782157900	-2.40092002204186
V	1.88075588746287	2.32667953304080	-1.15731005726761
O	2.51034190582926	3.65560236235029	-1.80362202365310
O	0.64645073480239	2.73115079703514	-0.01206254313658
O	0.63171508792924	-2.73919149641728	-0.05556375486053
V	1.86844080856247	-2.31733331857705	-1.19333062338951
O	1.18961277916657	-1.36482631891838	-2.42650443685757
O	2.49912469149629	-3.64057282897550	-1.85095008044953
O	1.04516459811861	-0.04527302783852	4.35690328898280
V	0.95841324086593	-0.02916418787966	2.75812815458815
O	0.11876355789438	1.39997281893835	2.27675729469800
O	0.11483883180917	-1.44680515486046	2.24935330977904
O	2.66661979016294	-0.02270217045242	2.12598062856645
O	5.09597146416312	-0.01739490256991	0.97925529257160
V	3.54349490344276	-0.00818303440278	0.60556559050228
O	3.23909467147082	-1.43915674594611	-0.35132524917990
O	3.25431386698517	1.44643053038393	-0.32194537530911
H	-3.73506947483268	4.26806104622539	1.87081830283970
H	-4.79387014200238	3.95666685203441	0.45702169859452
C	-4.24637461140099	3.49670948522562	1.28973108117535
C	-3.27260993517217	2.47589062104281	0.73816611355646

H	-4.98242789242145	2.97872800708427	1.91746048398158
O	-0.82361354496015	0.03173778838105	-3.55114500261261
O	-3.61294196511155	0.02794333620917	-2.20207029746616
